# Synthesis of some derivatives of 1,8-dioxo-octa-hydro xanthene and 9-aryl-hexahydro acridine-1,8-dione using metal ion-exchanged NaY zeolite as heterogeneous catalyst†

**DOI:** 10.1039/d3ra03020b

**Published:** 2024-03-28

**Authors:** Faeze Namayandeh Niasar, Mohsen Moradian

**Affiliations:** a Department of Organic Chemistry, Faculty of Chemistry, University of Kashan Kashan Iran; b Institute of Nanoscience and Nanotechnology, University of Kashan Kashan Iran m.moradian@kashanu.ac.ir

## Abstract

A method for synthesizing xanthene and acridine derivatives using transition metal catalysts supported by de-alumination zeolite-NaY is described. Some transition metal ion-exchanged NaY zeolite was prepared and evaluated in a reaction toward the synthesis of xanthene and acridine. The most active catalyst in this field is a heterogeneous copper/zeolite catalyst. The synthesis of xanthene with a wide range of aldehydes/dimedone with a molar ratio of 1/2 and acridine synthesis with a molar ratio of dimedone/aromatic/ammonium nitrate of 2/1/1 was carried out in one pot and solvent-free conditions. Sensing in the presence of supported metal catalysts is operationally simple, does not require expensive or toxic reagents, and gives high yields in a short period.

## Abstract 2

This is a process for producing xanthene and acridine derivatives. It uses copper metal catalysts supported by de-alumination zeolite-NaY. The process is conducted in a single pot and solvent-free conditions and does not require toxic reagents.

## Graphical abstract

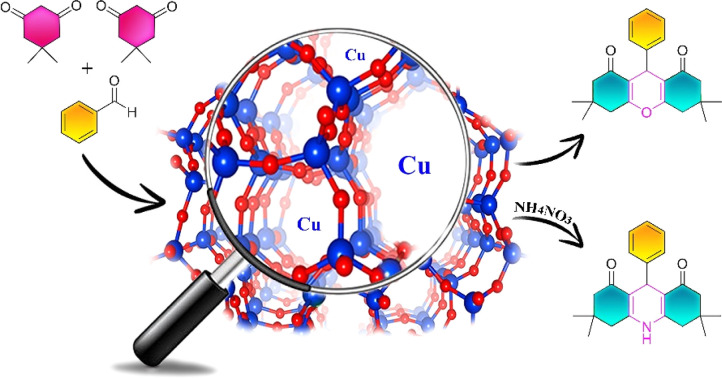

## 1 Introduction

Zeolites are compounds with a porous structure and large internal cavities that are composed of aluminium and silicate. Their origin is both natural and artificial. Extraction of aluminum from sodium zeolite framework Y was done for the first time by Kerr.^1^ He performed this process using ethylene diamine tetra-acetic acid solution at ambient temperature. In this article, 50% of aluminum was extracted from the zeolite without changing the structure of the primary zeolite, which is one of the most important advantages of this method.^1,2^ With the release of aluminum from the zeolite structure, the hydroxyl groups with acidic properties increase.^3^ End surface hydroxyl groups exist both in the form of pure silica and alumina, which is non-acidic.^4^ In 1964, Burke claimed the commercial synthesis of zeolite Y after Milton achieved the first industrial production of type A and X zeolites.^5,6^ Natural zeolites are rarely pure, so they are not used in many important commercial applications where uniformity and purity are important.^7^ Aluminum-rich zeolites are decomposed due to their instability in acid or water at high temperatures, without the framework collapsing.^8,9^ Exchangeable cations of a zeolite are cations that have a weak bond with the tetrahedral framework and are retained, these cations are easily exchanged by washing the zeolite with a stronger solution that has a different cation. It should be noted that zeolites do not perform anion exchange to any extent.^10^ Xanthenes are an important oxygen-containing ring compound whose main application is medicinal chemistry. Xanthene derivatives are essential due to their antibacterial, antiviral, anti-inflammatory and analgesic properties.^11^ They are also used differently as dyes in lasers.^12^ Acridines are a fundamental group of organic compounds because they have many medicinal and biological activities. Like the positive ionotropic effect that causes calcium to enter the intracellular space,^13^ they also have anticancer activity,^14^ enzyme and tumor cell inhibitors,^15^ antimicrobial activity, and cytotoxicity.^16^ Xanthenes and benzo xanthenes are synthesized by various methods, including trapping of types of gasoline by phenyls,^17^ and ring densities between 2-hydroxy-aromatic aldehydes and 2-tetralone.^18^ In addition, the synthesis of benzoxanthenes and related products includes the reaction of bethanechol with formamide,^19^ aldehyde, and cyclic compounds of 1,3-dicarbonyl. The preparation of acridines and their derivatives is a three-component reaction. This reaction is also part of an important group of organic reactions. They have medicinal properties such as the positive ionotropic effect which causes calcium to enter the cellular space.^20^ Acridines also have anticancer properties^21^ as well as being enzyme and tumor cell inhibitors.^22^ Furthermore, they have several antimicrobial and cytotoxic properties.^23^ Various methods for the synthesis of acridine derivatives containing 1,4-dihydropyridine from dimedone, aldehydes, and nitrogen sources namely urea,^24^ ammonium acetate in alumina^25^ ceric ammonium nitrate^26^ have been demonstrated in the past. Amines or ammonium acetate,^27^*via* conventional heating in organic solvents and in the presence of dodecylbenzene sulfonic acid,^28^ triethyl benzyl ammonium chloride,^29,30^ all while using *N*-arylidenenaphthalen-1-amine or *N*-arylidenequinolin-5-amine,^31^ trifluoroacetate 1-methyl imidazolium^32^ have also been previously demonstrated. In this study, we present a simple method for the synthesis of octahedral derivatives of 1,8-dioxoxanthene and 9-aryl-hexahydroacridine-1,8-dione using ion-exchange zeolite Y with metal cations as a heterogeneous catalyst. Karimi Rad *et al.* In 2020 glycerol-mediated and simple synthesis of 1,8-dioxo-decahydroacridines under transition metal-is described. The products have been synthesized from aromatic aldehydes and 5,5-dimethyl-1,3-cyclohexanedione (dimedone) with amines. Also, 1,8-dioxo-decahydroacridine derivatives are presented.^33^ Rocchi *et al.* in 2020 provided a three-component response constructed between chalcones, anilines, and beta-keto-esters followed by a microwave-assisted thermal cyclization afforded 1,3-diaryl-1,2-dihydroacridin-9(10*H*)-ones. Their microwave irradiation in nitrobenzene, acting both as solvent and oxidant, afforded fully unsaturated 1,3-diarylacridin-9(10*H*)-ones, which combine acridin-9-(10*H*)one and *m*-terphenyl moieties.^34^ In 2020, Kefayati *et al.* Synthesized benzoquinolone and benzo acridinone derivatives using an efficient magnetic nanocatalyst. Magnetic acid nanocatalyst for synthesizing of 2-amino-4-aryl benzoquinoline-3-carbonitrile and 10,10-dimethyl-7-aryl-9,10,11,12-tetrahydrobenzo acridin-8(7*H*)-one *via* alpha-naphthylamine and aromatic aldehydes were prepared with malononitrile or dimedone.^35^ In 2020, Daraie *et al.* performed the selective chemical synthesis of drug-like pyrrolo[2,3,4-*kl*] acridin-1-one using polyoxometalate@lanthanoid catalyst and dimedone, isatin and aniline under green conditions.^36^ In 2020, Kamat *et al.* prepared various derivatives of xanthene and coumarin with the help of beta-cyclodextrin as a reusable catalyst at 70 °C in water.^37^ Dikusar *et al.* In 2020 a suitable method for tetrahydrobenzo[*a*]acridine-11(7*H*)-one and dihydrobenzo[*f*]pyrimido[4,5-*b*]quinoline-9,11(7*H*,8*H*)-dione containing residues of nicotinic and isonicotinic acids covalently attached *via* ester groups in different positions of the aromatic core. Quaternary ammonium salts of the synthesized acridine derivatives as well as a metal complex with palladium PdLCl_2_ were obtained.^38^ Liu *et al.* In 2020 a new series of pyrazino[2,3-*a*]acridine derivatives were prepared, *via* three-component reaction of quinoxaline-6-amine, aromatic aldehydes, and 5,5-dimethyl cyclohexane-1,3-dione or cyclohexane-1,3-dione in ethanol as a solvent and under rehabilitation conditions.^39^ In 2021, Mehrabadi *et al.* was synthesized *via* preparing pistachio hull as a support followed by treatment with titanium tetrachloride (TiCl_4_) as a nanocatalyst in a one-pot, three-component condensation reaction of aromatic aldehydes, dimedone and kojic acid in refluxing ethanol to furnish dihydropyrano[3,2-*b*]chromendione derivatives.^40^ In 2021, Sanai Rad *et al.* performed a multicomponent reaction of aromatic aldehydes, aryl amines, malononitrile, and dimedone to synthesize hexahydroquinolines derivatives in the presence of a strong catalyst.^41^ In 2021 Patel *et al.* Synthesis of spiro[indoline-3,9′-xanthene] trione and spiro[chromene-4,3′-indoline]-3-carbonitrile derivatives using graphene oxide-supported dicationic ionic liquid as a heterogeneous catalyst in aqueous media.^42^ In 2021, Chesnokov *et al.* Obtained acridin-4-ols reaction with the alkylation of anilines with 3,5-di-*tert*-butyl-6-methoxymethylcatechol, followed by oxidation of the mixture.^43^ In 2021, Sam *et al.* Investigated a catalytic activity for the synthesis of poly-hydro acridines as well as poly hydro quinolines through reactions of dimedone or ethyl acetate, various aldehydes, and ammonium acetate in ethanol.^44^ In 2021, Nami *et al.* Synthesized spiro-acridine/indoline and indoline derivatives by the three-component reaction of isatin, dimedone, and amines or amino acids in the presence of acid functionalized multi-walled carbon nanotubes as a catalyst in ethanol.^45^ In 2021, Fekri *et al.* Used the effective copper/dapsone nanocomposite of propyl covalent for the multi-component reaction of aldehydes, dimedone and dapsone to synthesize acridines.^46^ In 2021, one-pot three-component cyclo-condensation reaction of bis(indole-2,3-diones) with dimedone, 3-methyl-1*H*-pyrazol-5(4*H*)-one, or 6-aminouracil in boiling acetic acid afforded bis-spirocyclic oxindoles linked to acridine, dipyrazolo[3,4-*b*:4′,3′-*e*]pyridine, and pyrido[2,3-*d*:6,5-*d*′]dipyrimidine, respectively.^47^

**Scheme 1:**
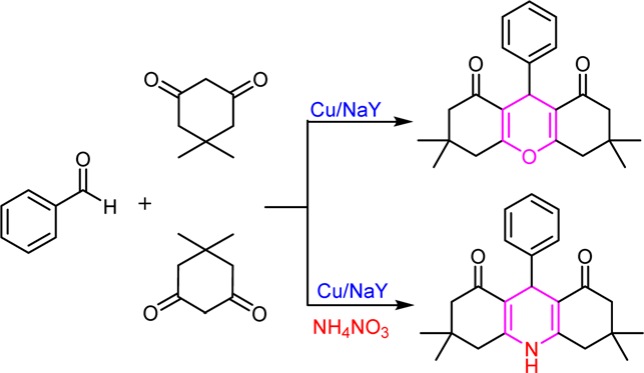
Prepared xanthene and acridine in the presence of Cu/NaY.

In continuation of our studies in the field of solid catalysts,^48^ transition metal ion-exchanged Na–Y zeolite was synthesized and characterized using spectroscopic methods. The synthesized catalyst was used in the reaction of dimedone, aromatic aldehydes, and ammonium nitrate toward acridines and xanthene derivatives (Scheme 1). The synthesized acridine and xanthenes were separated and purified. Their structures were investigated and identified by infrared spectroscopy and hydrogen nuclear magnetic resonance analysis methods. The synthesized M(ii)–NaY catalyst has a cavity that results in a large active surface area. This feature increased the yield of the products and reduced the reaction time, indicating the efficiency of this catalyst in making the obtained compounds. The present method is operationally simple, highly efficient, solvent-free, has a short reaction time, and uses inexpensive catalysts.

## 2 Experimental

### 2.1. Materials

Chemicals such as various benzaldehydes, dimedone, ammonium nitrate, copper nitrate, cobalt nitrate, nickel nitrate, and manganese nitrate were provided from Floka, Merck, and Asia Research companies with high purity. Ethanol, acetone, ethyl acetate, petroleum ether, and tetrahydrofuran solvents were also obtained from Asia Research and Merck, Germany and was used without extra purification.

### 2.2. Instrumental measurements

To identify the product in these studies, the magnetic resonance devices of the hydrogen core model 400 DRX of 400 MHz broker were doped in dimethyl sulfoxide solvent, and tetra-methyl-silane (TMS) was made as an internal control in the Kashan University environment. In this study, the spectra were prepared by FT-IR device made by Nicolette company and Magna 550 type using potassium bromide tablets in Kashan University. Also, for SEM analysis with FEIESEM QUANTA 200_EDAX SILICON DRIFT 2017 specifications and XRD device with used specifications, model: X'PertPro, made in the Netherlands, company: PANalytical used.

### 2.3. Catalytic tests

#### 2.3.1. Process for de-alumination of NaY

In this study, a de-aluminium method was used to activate the zeolite. 0.5 g of Na–Y zeolite with 7 ml of ion-free water and 0.028 g of ethylene diamine tetra-acetic acid were refluxed at 100 °C for one hour. The smooth reaction mixture was washed with hot water and dried at 160 °C for 2.5 hours. With this method, almost all the aluminium in Na–Y zeolite is removed, and its vacancy is prepared as an active part for accepting ions.

#### 2.3.2. Preparation of Cu/NaY

Initially, 3.2 mmol of 3-aqueous copper nitrate (blue crystal), equivalent to 0.77 g, was dissolved in 400 ml of deionized water in a 500 ml flask at reflux and ambient temperature. Then 20 g of NaY zeolite was added and stirred for 24 hours at room temperature. Then it was filtered, and the material on the filter was washed twice with ion-free water and dried for 12 hours at 120 °C.

#### 2.3.3. Preparation of Co/NaY

To prepare cobalt zeolite, 3.2 ml of 6-aqueous cobalt nitrate equivalent to 0.93 g, which is red in color, was added to 400 ml of deionized water in a 500 ml flask under reflux conditions. After the metal salt was dissolved, 20 g of NaY zeolite was added and refluxed for 24 hours. The mixture was then reacted with ion-free water and dried at 120 °C.

#### 2.3.4. Preparation of Ni/NaY

Nickel zeolites were mixed by mixing 3.2 ml of 0.93 g of aqueous nickel nitrate 6 (emerald green solid) with 400 ml of deionized water in a 500 ml flask under reflux conditions. After dissolving the metal salt, 20 g of NaY zeolite was added to the mixture and refluxed together for 24 h. The reaction mixture was then filtered and washed well with ion-free water and dried at 120 °C.

#### 2.3.5. Preparation of Mn/NaY

6-aqueous manganese, which is a white crystalline solid at the rate of 3.2 mmol (0.28 g), along with 400 ml of ion-free water in a 500 ml flask, is liquefied until the metal salt is dissolved, then 20 g of zeolite is added to the mixture and mixed for 24 hours at room temperature. The mixture was then filtered and washed well with ion-free water and dried at 120 °C. It should be noted that XRD and SEM analysis methods were used to analyze and prepare copper, cobalt, nickel, and manganese zeolites.

#### 2.3.6. Preparation of xanthene derivatives

2 mmol of dimedone and 1 mmol of aromatic benzaldehyde, along with 0.02 g of a Cu/NaY catalyst, were placed at 110 °C without solvent. The reaction mixture was stirred for several minutes. After considering the development of the reaction, by thin-layer chromatography, the mixture was dissolved in tetrahydrofuran solvent so that the zeolite catalyst could be easily removed from the reaction medium. The mixture is then poured into an ice bath and mixed well. The resulting precipitate is filtered and washed well with distilled water and dried at room temperature.

#### 2.3.7. Preparation of acridine derivatives

2 mmol of dimedone, 1 mmol of aromatic benzaldehyde, 1 mmol of ammonium nitrate and 0.02 g of Cu/NaY catalyst were placed in solvent-free at 110 °C for several minutes. After considering the development of the reaction, the mixture was dissolved in tetrahydrofuran solvent by thin-layer chromatography so that the Cu/NaY catalyst could be easily removed from the reaction medium. Then the mixture was poured into an ice bath and the resulting precipitate was washed smoothly and well with distilled water and dried at room temperature.

## 3 Results and discussion

### 3.1. Synthesis and characterization of M(ii)/NaY

**Fig. 1:**
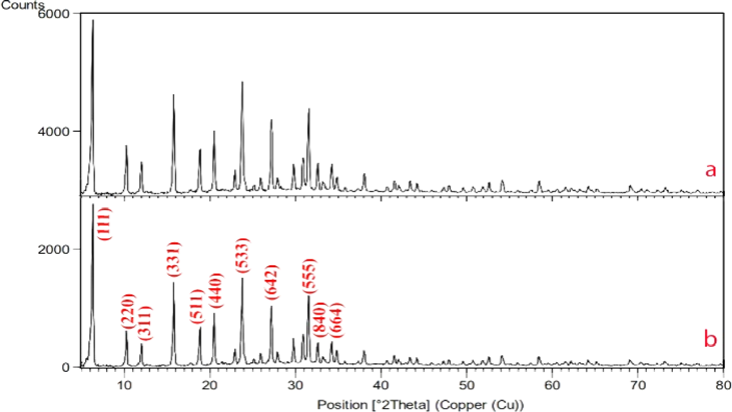
XRD of the zeolite before de-aluminium (a), XRD zeolite after de-aluminium (b).

Zeolite needs to be activated to prepare for the reaction and cation exchange within its system, which is possible by removing aluminum from within the zeolite system. By removing aluminum from the zeolite structure, a space is created that allows the exchange of cations in the zeolite structure, and the cation can be more easily placed inside the zeolite. That is why in the image we took from the sample before and after de-aluminum, the structure of zeolite was not changed and destroyed and its original nature and framework were preserved. Here the XRD diagram before and after de-aluminum is recorded in Fig. 1.

As can be seen, in the range 0–5 in Fig. 1(a) and (b) there are strong and long peaks that are similar in both images. Other peaks can be seen in both forms without significant change.

**Fig. 2:**
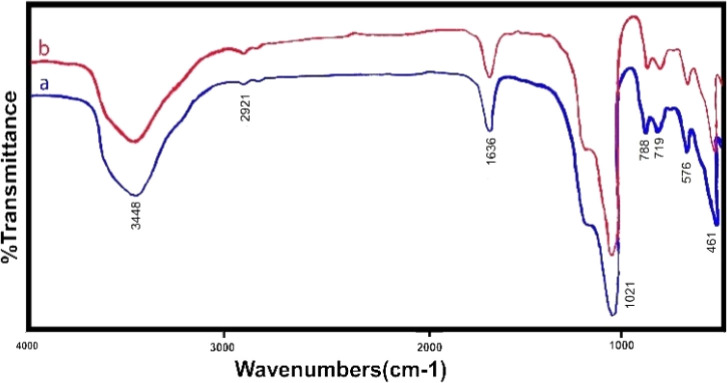
Infrared spectrum of zeolite: (a) zeolite before de-aluminum, (b) zeolite after de-aluminum.

Various analysis methods were used to study the structure. Fig. 2 shows the infrared analysis of zeolite before and after de-aluminum. As shown in the figure, the spectrum is blue, zeolite before de-aluminum and the spectrum is red, zeolite after de-aluminum. No significant changes are observed in the spectra, which means that the main structure of the zeolite does not change.

**Fig. 3:**
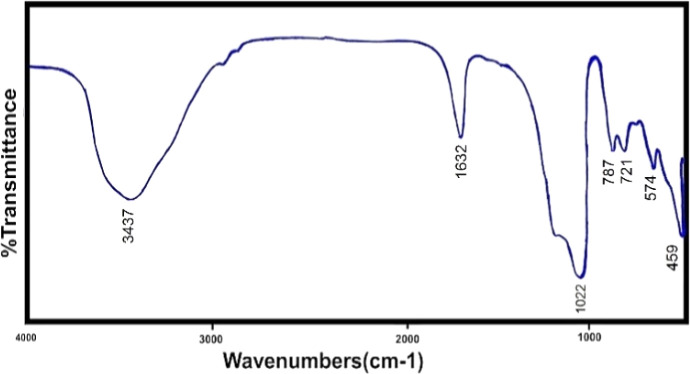
Infrared spectrum of cation-exchanged zeolite with manganese metal.

The FTIR spectrum shows two intense bands at 576 cm^−1^ and 719 cm^−1^, which correspond to the stretching vibration of Si–O and Al–O, respectively. The bands at 3448 cm^−1^ are attributed to O–H stretch vibration of hydroxyl groups and 1636 cm^−1^ to the bending state (H–OH). In addition, the peak appearing at 461 cm^−1^ corresponds to the bending vibration (M–O) of the metals that have been incorporated. The stretching vibrational frequencies around 2921 and 1021 cm^−1^ correspond to atmospheric or adsorbed CO_3_ and nitrate ions, respectively. Also shown in Fig. 3 is the infrared spectrum of ion-exchanged zeolite with manganese metal, which is clear that only manganese metal replaces another metal in the structure and causes minor changes within the structure.

**Fig. 4:**
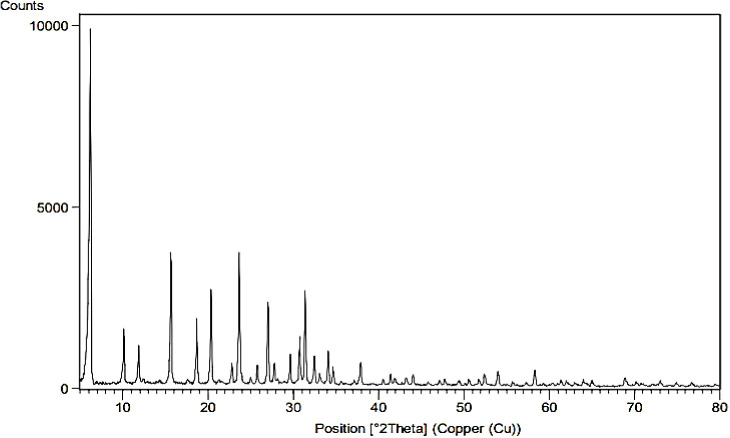
XRD ion-exchange zeolite with manganese metal.

It is also clear from the Fig. 4 that the manganese zeolite spectrum has not changed significantly from the original zeolite spectrum and only the intensity of the spectra has changed. In cation exchange, manganese metal replaces sodium metal, and certainly, 100% of sodium is not removed from the structure and some of it remains inside the system.

**Fig. 5:**
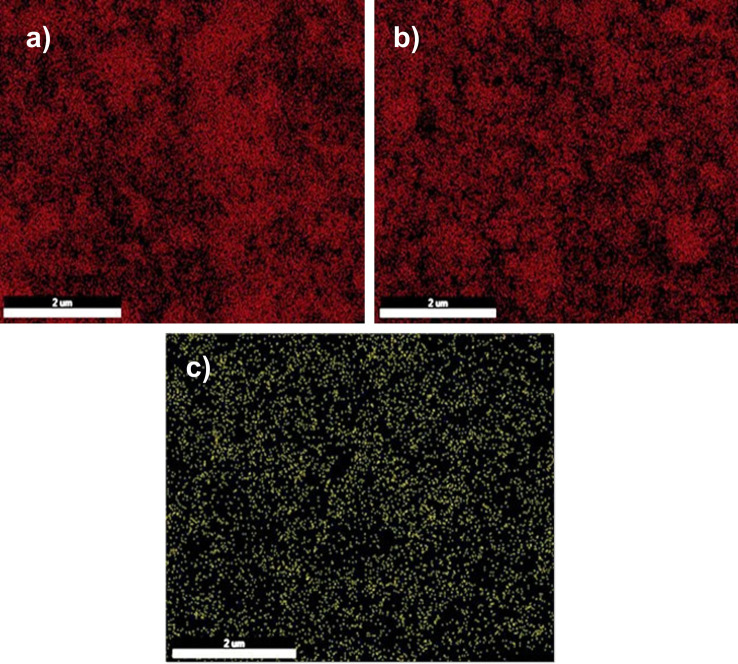
Abundance of silicon in de-aluminum zeolite (a), abundance of copper in Cu/NaY (b), abundance of manganese in manganese zeolite (c).

We investigated the structure of the decomposed aluminum zeolite, Cu/NaY, and manganese zeolite was analyzed by EDAX. The images in Fig. 5 show the frequency of the relevant elements.

X-ray fluorescence (XRF) is a non-destructive analytical technique used to determine the elemental composition of materials. The results for the composition of the catalyst Cu(ii)/NaY are shown in Table 1.

**Table 1:** XRF analysis of the catalyst Cu(ii)/NaY

Oxides	SiO_2_	Al_2_O_3_	Na_2_O	Cu	MgO	CaO	Other oxides
W%	38.23	11.82	31.17	3.91	1.84	2.05	10.98

### 3.2. Catalytic properties of studied Cu(ii)/NaY

**Scheme 2:**
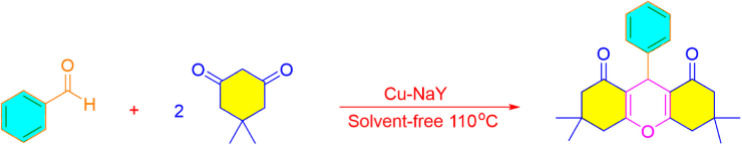
Use of Cu/NaY in the synthesis of xanthene.

The prepared Cu/NaY was used as the active catalyst for xanthene synthesis. In the continuation of this study, first, the reaction was performed at 110 °C from dimedone with aromatic aldehydes in the presence of a Cu/NaY catalyst (Scheme 2).

#### 3.2.1. Optimization of the amount of Cu/NaY catalyst in the preparation of xanthene derivatives

The reaction of benzaldehyde (1 mmol) and dimedone (2 mmol) was performed in the presence of Cu/NaY at 110 °C. The reaction was repeated several times to optimize the amount of catalyst. The values obtained in Table 2 show that if 2 mg of copper zeolite catalyst is used, the maximum gain is obtained in 3 minutes.

**Table 2:** Optimization of Cu/NaY catalyst in xanthene synthesis^a^

Reaction number	Amount of catalyst (mg)	Time (minutes)	Yield (percentage)
1	0	45	25
2	1	10	72
3	2	3	98
4	3	3	98

a Reaction conditions: 2 mmol of dimedone, 1 mmol of benzaldehyde, 110 °C.

#### 3.2.2. Optimization of the reaction temperature of xanthene preparation using Cu/NaY catalyst

The reaction of benzaldehyde (1 mmol) and dimedone (2 mmol) was performed in the presence of Cu/NaY catalyst (2 mg) at different temperatures and repeated several times to determine the optimum temperature. The values recorded in Table 3 indicate that the ideal temperature for the reaction is 110 °C.

**Table 3:** Optimization of xanthene synthesis reaction temperature in the presence of Cu/NaY catalyst^a^

Row	Temperature (degrees celsius)	Time (minutes)	Yield (percentage)
1	25 °C	40	20
2	80	30	50
3	100	20	70
4	110	3	98
5	120	4	98

a Reaction conditions: 2 mmol of dimedone, 1 mmol of benzaldehyde and 2 mg of Cu/NaY catalyst.

### 3.3. Efficiency of Cu/NaY recycling test

**Fig. 6:**
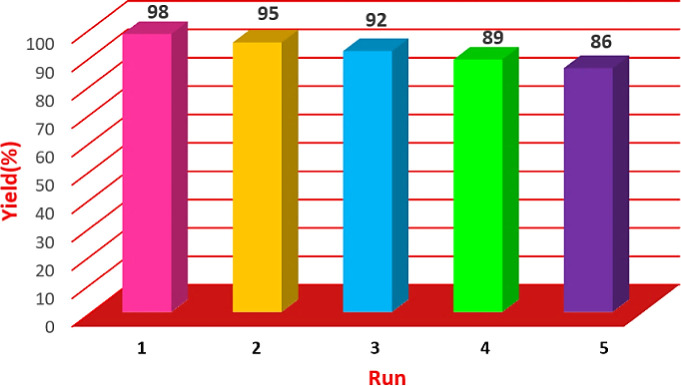
Investigation of catalyst recyclability.

The catalyst used was evaluated for recovery after optimization of the reaction conditions. The Cu/NaY catalyst in the xanthine preparation reaction was separated from the reaction mixture by refining and washed and drying 2 to 3 times with ethanol and reusing. Catalyst recovery rate and product efficiency were recorded in Fig. 6. As shown in the table, even after 5 times of recycling of the catalyst, its efficiency is still significant.

#### 3.3.1. Investigation of the effect of M(ii)/NaY catalysts in the preparation of xanthene

To prepare xanthenes, 4 types of ion exchange zeolites of copper, cobalt, manganese, and nickel were prepared and used. The catalysts were each used separately in the reaction of benzaldehyde (1 mmol) and dimedone (2 mmol) at 110 °C without solvent to prepare xanthan. Cu/NaY as a catalyst had the highest efficiency among other catalysts. Other catalysts were somewhat successful in the production process. The efficiency of catalysts is recorded in Table 4.

**Table 4:** Evaluation of the efficiency of ion exchange zeolite catalysts in the preparation of xanthenes^a^

Test number	Catalyst	Weight percent (%) of selected metal	Yield (percentage)
1	Cu/NaY	3.91	98
2	Co/NaY	3.88	57
3	Ni/NaY	3.90	40
4	Mn/NaY	3.81	47

a Reaction conditions: 2 mmol of dimedone, 1 mmol of benzaldehyde and 2 mg of catalyst, 110 °C.

#### 3.3.2. Results of preparation of xanthens in the presence of Cu/NaY catalyst

First, the reaction between dimedone and benzaldehyde derivatives was carried out in the presence of the Cu/NaY catalyst. For this purpose, one mmol of benzaldehyde derivative and two mmol of dimedone were exposed to solvent-free conditions at 110 °C in the presence of 2 mg of Cu/NaY catalyst. When the reaction was complete, it was dissolved in tetrahydrofuran to separate the catalyst. An ice bath was then used to form a precipitate. The resulting precipitate was thoroughly washed with water and dried at room temperature. The results of the products are recorded in Table 5.

**Table 5:** Product and reaction efficiency of formation of different derivatives using Cu/NaY catalyst in heat and without solvent

Entry	Structure of products (acridine product code)	Time (min)	Isolated yields (%)	M. P. (°C)	
				Observed	Reported
1	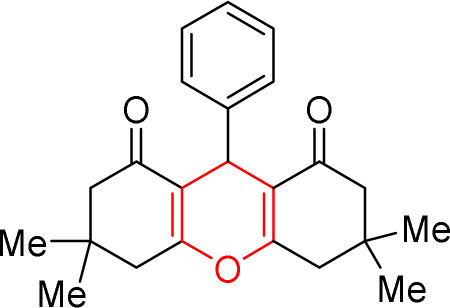	2	98	194–198	202–204
2	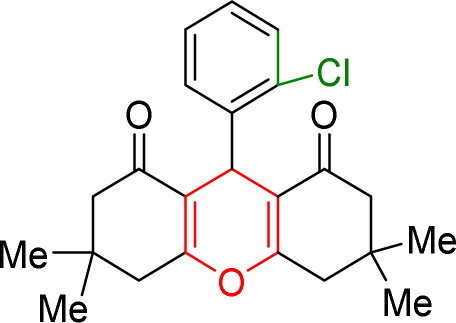	3	97	224–225	226–227
3	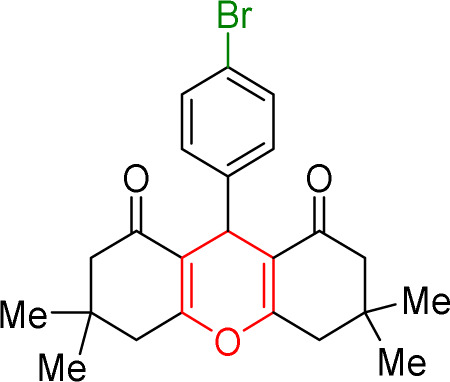	2	98	260–262	261–262
4	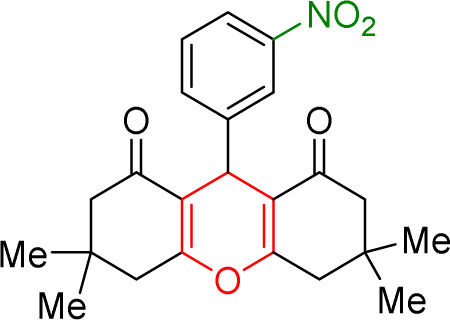	4	97	166–170	166–168
5	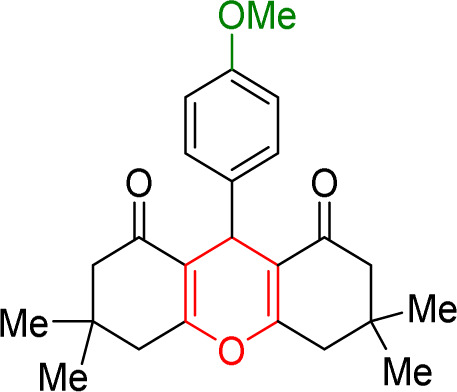	4	96	241–243	245–246
6	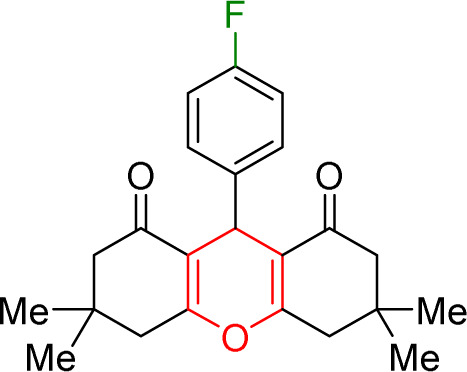	3	93	223–224	225–226
7	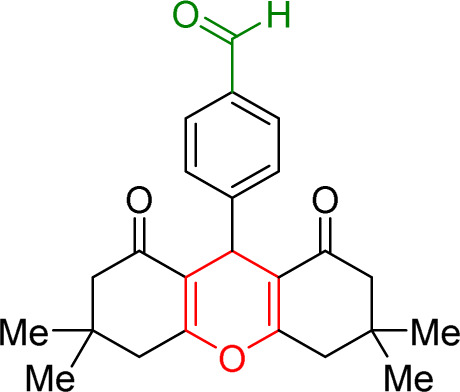	4	95	210–212	211–213
8	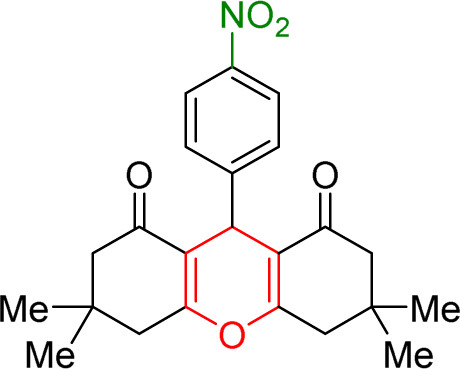	2	97	220–222	221–222

### 3.4. Reaction mechanism

**Scheme 3:**
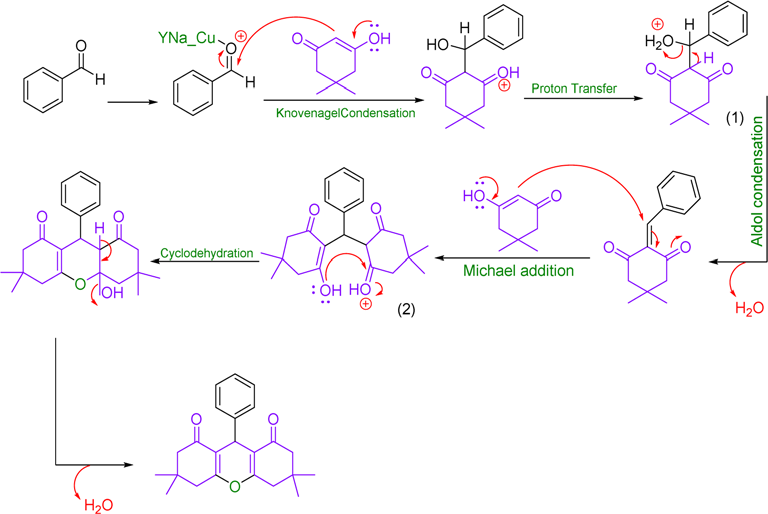
Plausible reaction mechanism for the synthesis of 1,8-dioxo-octa-hydro xanthene.

Concentration between various benzaldehydes with dimedone and strong acid as a catalyst is a common for xanthine synthesis. In the lead reaction, the role of the catalyst is vital because the activation of carbonyl carbon in aldehyde is required to start the reaction. Therefore, in the mechanism, first, a pair of carbonyl oxygen electrons are placed in the empty orbital of the catalyst so that with this bond, benzaldehyde becomes an appropriate electron friend and the reaction continues. Then, the first molecule of dimedone, which is in an equilibrium of ketone enol with its acidic hydrogen, through its acidic hydrogen, performs a compression reaction of Knoevenagel with benzaldehyde, and after a proton exchange step, creates an intermediate (1). Slowly dimedone acidic hydrogen, which is in a good position relative to ^+^OH_2_ benzaldehyde, helps to drain water and builds up aldol in the system. Then, with the addition of the second molecule of dimedone, an intermediate (2) is created, which is the result of Michael's reaction. The system helps and xanthine forms (Scheme 3).^49^

### 3.5. Use of Cu/NaY in the reaction of preparation of acridines

Cu/NaY was used as the active catalyst for the synthesis of acridine. At the beginning of the study, acridine was prepared from three-component concentrations of dimedone, aromatic aldehydes, and ammonium nitrate in the presence of Cu/NaY catalyst at 110 °C.

#### 3.5.1. Optimization of Cu/NaY catalyst in the preparation of acridine derivatives

**Scheme 4:**
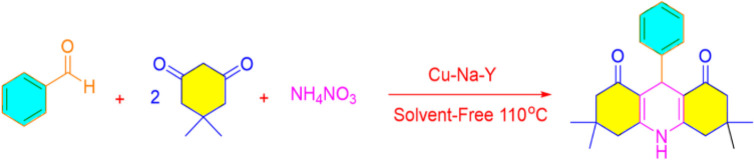
Use of Cu/NaY in the synthesis of acridines.

The reaction of benzaldehyde and dimedone and ammonium nitrate was performed in the presence of Cu/NaY at 110 °C. The reaction was repeated several times to optimize the amount of catalyst. The values obtained in Table 6 show that if 2 mg of Cu/NaY catalyst is used, the maximum gain is obtained in 3 minutes (Scheme 4).

**Table 6:** Optimization of Cu/NaY catalyst in the synthesis of acridines^a^

Reaction number	Amount of catalyst (mg)	Time (min)	Yield (%)
1	0	45	5
2	1	11	60
3	2	3	97
4	3	3	97

a Reaction conditions: 2 mmol of dimedone, 1 mmol of benzaldehyde, 1 mmol of ammonium nitrate, 110 °C.

#### 3.5.2. Optimization of reaction temperature of acridines prepared using Cu/NaY catalyst

The reaction of benzaldehyde and dimedone and ammonium nitrate in the presence of Cu/NaY catalyst was performed at different temperatures and repeated several times to determine the optimum temperature. The values recorded in Table 7 indicate that the best temperature for the ideal reaction is 110 °C.

**Table 7:** Optimization reaction temperature of acridine synthesis in the presence of Cu/NaY catalyst^a^

Row	Temperature (°C)	Time (min)	Yield (%)
1	25	30	25
2	80	30	50
3	100	25	70
4	110	3	98
5	120	3	98

a Reaction conditions: 2 mmol of dimedone, 1 mmol of benzaldehyde, 1 mmol of ammonium nitrate, 110 °C.

### 3.6. Investigation of recycling rate of Cu/NaY catalyst

**Fig. 7:**
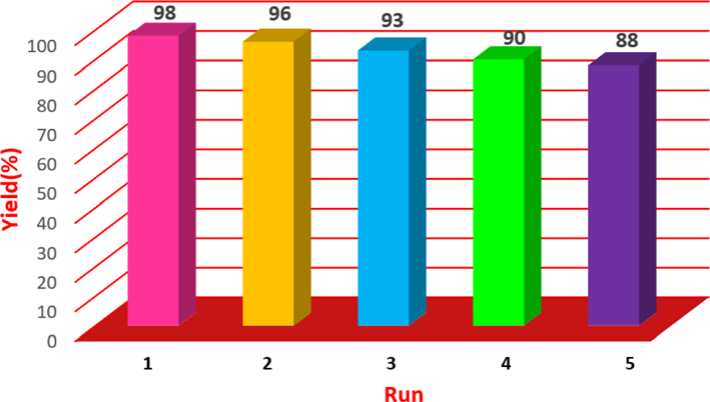
Investigation of catalyst recyclability.

The catalyst was tested for recyclability after optimizing the reaction conditions. The Cu/NaY catalyst in the reaction was separated from the reaction mixture by filtration. It was then washed and dried 2 to 3 times with ethanol and reused. Catalyst recovery achievement and product efficiency are recorded in Fig. 7. As shown in the table, the reaction can still be performed after reusing the recycled catalyst 5 times.

#### 3.6.1. Investigation of the effect of ion exchange zeolite catalysts

In this study, 4 types of ion exchange zeolites of copper, cobalt, manganese, and nickel were prepared. Each of the catalysts was used separately in the acridine preparation reaction, of which the Cu/NaY catalyst in the reaction of benzaldehyde, dimedone, and ammonium nitrate had the highest efficiency among the other catalysts. Other catalysts also led to the production of the product, the efficiency of which is recorded in Table 8.

**Table 8:** Evaluation of the efficiency of ion exchange zeolite catalysts^a^

Test number	Catalyst	Weight percent (%) of selected metal	Yield (%)
1	Cu/NaY	3.91	97
2	Co/NaY	3.88	51
3	Ni/NaY	3.90	47
4	Mn/NaY	3.81	50

a Reaction conditions: 2 mmol of dimedone, 1 mmol of benzaldehyde, 1 mmol of ammonium nitrate and 2 mg of catalyst, 110 °C.

#### 3.6.2. Results of preparation of acridines in the presence of Cu/NaY catalyst

In acridine synthesis, benzaldehyde, dimedone, and ammonium nitrate derivatives were extracted in the presence of Cu/NaY catalyst under solvent-free conditions at 110 °C and the product was extracted. The results of the products are recorded in Table 9.

**Table 9:** Product and reaction efficiency of formation of different acridine derivatives using heat-treated and solvent-free Cu/NaY catalyst^a^

Entry	Structure of products (acridine product code)	Time (min)	Isolated yields (%)	M. P. (°C)	
				Observed	Reported
1	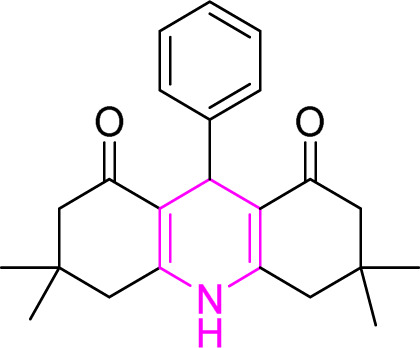	3	97	244–246	246–248
2	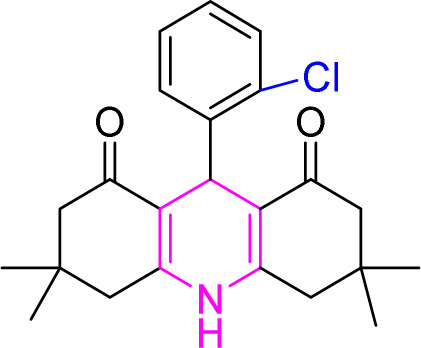	2	97	289–291	288–290
3	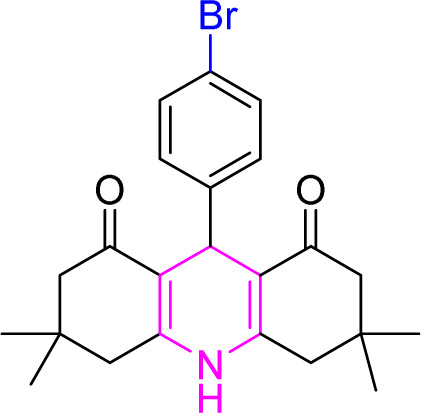	1	98	234–235	233–235
4	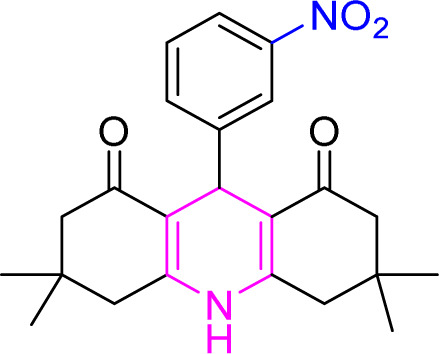	3	98	268–270	273–275
5	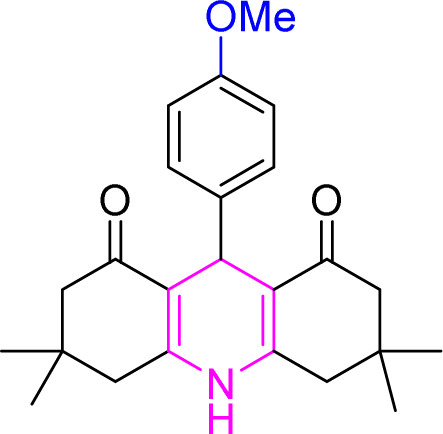	1	96	301–303	298–300

a Reaction conditions: 2 mmol of dimedone, 1 mmol of benzaldehyde, 1 mmol of ammonium nitrate and 2 mg of Cu/NaY catalyst, 110 °C.

### 3.7. Plausible reaction mechanism for the synthesis of 9-aryl-hexahydro acridine-1,8-dione

**Scheme 5:**
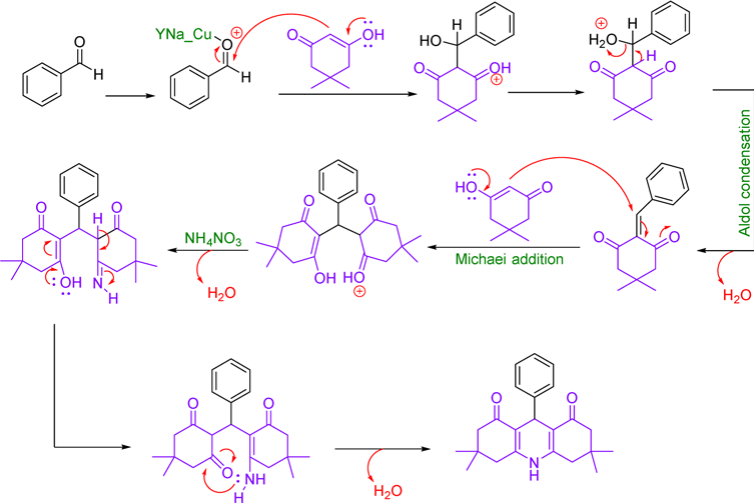
Plausible reaction mechanism for the synthesis of 9-aryl-hexahydro acridine-1,8-dione.

In this mechanism, as in the preparation of xanthene, the catalyst plays a major role in initiating the reaction, which is followed by the addition of the first molecule of dimedone to the system, after the exchange of water cations, but with the difference that following the addition of the second molecule of dimedone, the ammonium molecule nitrate enters the system and after the water leaves, it attacks the carbonyl dimedone with its electron pair, and this time with the withdrawal of water, acridine is obtained (Scheme 5).^50^

### 3.8. Investigation of leaching test

To better evaluate the catalyst's performance, this method examined the catalyst leaching. Once the half-reaction time was reached, the Cu(ii)/NaY solid catalyst was separated from the media by centrifugation. The reaction was then monitored in the solution phase without any fresh catalyst under identical conditions. After 3 hours, thin-layer chromatography (TLC) analysis revealed that the reaction had not progressed and failed to convert the substrates into the desired product. This indicates that neither the solid Cu(ii)/NaY catalyst nor its active metal leached into the filtrate liquor. To further investigate, atomic absorption spectroscopy analysis of the filtrate liquor demonstrated negligible copper leaching from the solid catalyst.

## 4 Conclusions

As a result, copper was included in the structure of zeolite Y. Used as a catalyst in the reaction of 1,8-dioxo-octa-hydro xanthene and 9-aryl-hexahydro acridine-1,8-dione. It is remarkable for it to react in a short time without using a solvent. Easy preparation of the catalyst, short reaction time, and purification of the product without the need for chromatography are the highlights of this research. Also, the catalyst still performs well after several uses.

## Conflicts of interest

There are no conflicts to declare.

## Supplementary Material

RA-014-D3RA03020B-s001
