# Supplementary material for: Synthesis of some derivatives of 1,8-dioxo-octa-hydro xanthene and 9-aryl-hexahydro acridine-1,8-dione using metal ion-exchanged NaY zeolite as heterogeneous catalyst
Source: RSC Adv. 2024 Mar 28;14(15):10322–30. doi: 10.1039/d3ra03020b (PMC10977046; doi:10.1039/d3ra03020b)
Supplement: RA-014-D3RA03020B-s001 [file RA-014-D3RA03020B-s001.pdf]

Electronic Supplementary Material (ESI) for RSC Advances.
This journal is © The Royal Society of Chemistry 2024

Supporting Information

Synthesis of some derivatives of 1,8-dioxo-octa-hydro xanthene and 9-aryl-hexahydro acridine-1,8-dione using metal ion-exchanged NaY zeolite as heterogeneous catalyst

Faeze Namayandeh Niasar, and Mohsen Moradian*

Department of Organic chemistry, Faculty of Chemistry, University of Kashan, Kashan, Iran.

Institute of Nanoscience and Nanotechnology, University of Kashan, Kashan, Iran

Corresponding author E mail: m.moradian@kashanu.ac.ir

Spectral investigation and structure description of 9- phenyl-3,3,6,6-tetramethyl-1,2,3,4,5,6,7,8-octa-hydro-xanthene-1,8-dione

Xanthen compounds were analyzed after preparation, separation, and purification to confirm the structure. In this section, a spectrum was selectively examined. Infrared spectra of the compound 9-phenyl-3,3,6,6-tetramethyl-1,2,3,4,5,6,7,8-octa-hydro-xanthene-1,8-dione in **Fig.1** Confirms the tensile vibration of C-H, sp^3 in the region ῡ = 2955 cm^{-1}. The strong message appeared in the region ῡ = 1633 cm^{-1} and ῡ = 1364 cm^{-1} showed the tensile vibration of the C = C bond which corresponds to the aromatic ring, the tensile vibration of C-O-C in the region ῡ = 1151-1199 cm^{-1} which Confirms the formation of the product. It also confirms sp^2 in the area of ῡ = 698 cm^{-1} and ῡ = 1005 cm^{-1} the off-plane flexural absorption of C-H confirms sp^2.

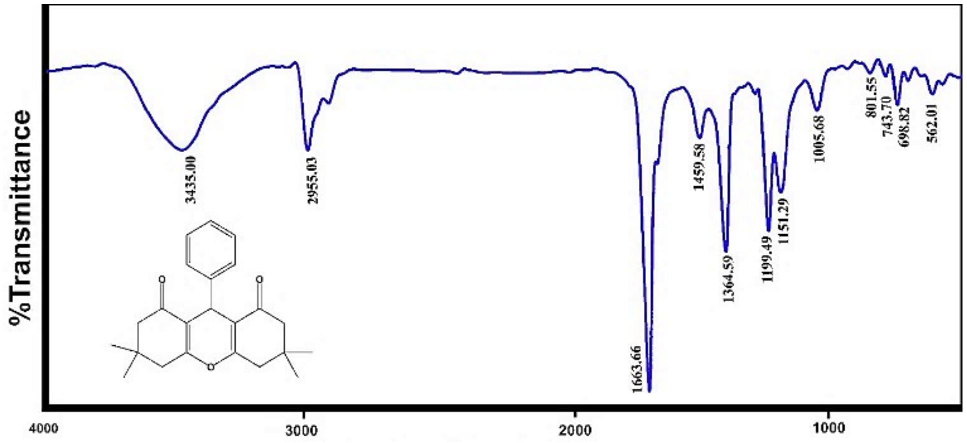

Fig.1- Infrared spectrum of compound ¬9- Phenyl-3,3,6,6-¬tetramethyl-1,2,3,4,5,6,7,8-octa-hydro-xanthene-1,8 – dione

Across ^{1}HNMR in **Fig.2** shows that methyl group hydrogens in the aliphatic region show δ = 0.88 and δ = 1.02, two unique messages. In the region of δ = 4.50, a single message is observed which indicates the formation of the product and is related to benzene hydrogen, which is affected by the anisotropy of benzene rings and is subject to high chemical displacement and lower field. Also, multiple fissures in the range of δ = 7 . 07-7 . 22 are aromatic ring hydrogens that have manifested themselves. In the range of δ = 2.04 - 2.59, the fissures of the methylene group also show that of the two hydrogens next to the oxygen of the xanthine ring, one of them appears in the region of δ = 2.23 - 2.27 in dual form. Another is revealed in the area δ = 2-04-04-08 in binary form. The two hydrogens adjacent to the carbonyl group also become more uncovered due to the lethal effect of the carbonyl and appear quadratically in the lower field δ = 2.49 - 4.59.

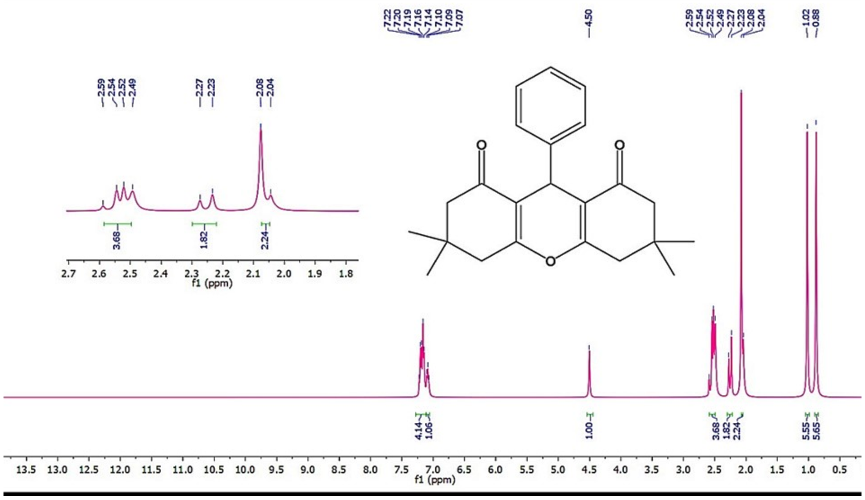

Fig.2- 1HNMR spectrum of compound ¬9- Phenyl-3,3,6,6-¬tetramethyl-1,2,3,4,5,6,7,8-octa-hydro-xanthene-1,8 -dione

Histological examination and description of the synthetic structure of 9-phenyl-3,3,6,6-tetramethyl-3,4,6,7,9,10-hexa-hydro-acridine-1,8-dione

The prepared acridine was analyzed after separation and purification to confirm the structure. In this section, one is examined and described.

Fig.3 shows the infrared spectrum of the compound 9-phenyl-3,3,6,6-tetramethyl-3,4,6,7,9,10-Hexa-hydro-acridine-1,8-dione. In the region of ῡ= 750 cm^{-1} and ῡ= 1007 cm^{-1} the extrinsic flexural absorption of C-H, sp^2 ortho-split aromatic rings of ortho type is shown. The tensile vibration of C-H, sp^3 appeared in the region of ῡ= 2958 cm^{-1}, also the strong vibration in the region of ῡ= 1665 cm^{-1} and ῡ= 1356 cm^{-1} is related to the C = C bond of the aromatic ring, and finally confirms the product that In the region ῡ= 1205 cm^{-1} and ῡ= 1157 cm^{-1}tensile vibration C-N. N-H tensile adsorption also appeared in the area of ῡ= 3566 cm^{-1}, which was flattened due to moisture.

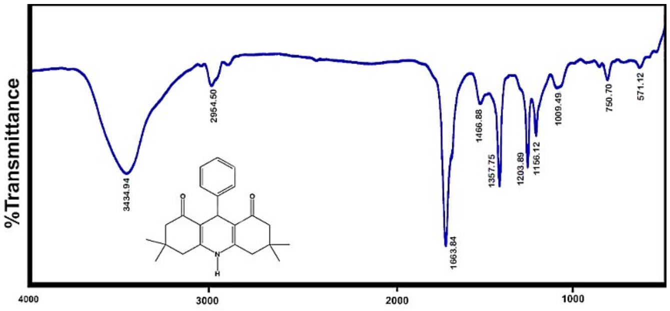

Fig.3- Infrared products of 9-phenyl-3,3,6,6-tetramethyl-3,4,6,7,9,10-hexa-hydro-acridine-1,8-dione

The 1H NMR spectra of the 9-phenyl-3,3,6,6-tetramethyl-3,4,6,7,9,10-Hexa-hydro-acridine-1,8-dione are shown in **Fig.4**. In the region of δ = 0.87 and δ = 1.01, there are two unique messages, each representing six hydrogens related to the fission of methyl hydrogens. In the region of δ = 4.50, there is a single message related to benzyl hydrogen and the observed band in the region of δ = 3.58 is related to hydrogen attached to nitrogen, which is correct with the presence of deuterium water according to Figure 9. Approved and indicates product formation.

Also, the messages in the range δ = 7. 08 -7. 20 are related to the five hydrogens of the aromatic ring. The fissures of the methylene group are also in the aliphatic range in the form of binary bonds corresponding to two hydrogens adjacent to the nitrogen, one of which is on the same plane with the electron-nitrogen pair and goes to the lower field, ie δ = 2.23-2.27. Another appeared in the upper field, δ = 2.04 - 2.07, and also two hydrogens adjacent to the carbonyl, which were affected by its lethality, and in the lower field, the range δ = 2.52 - 2.54 appeared.

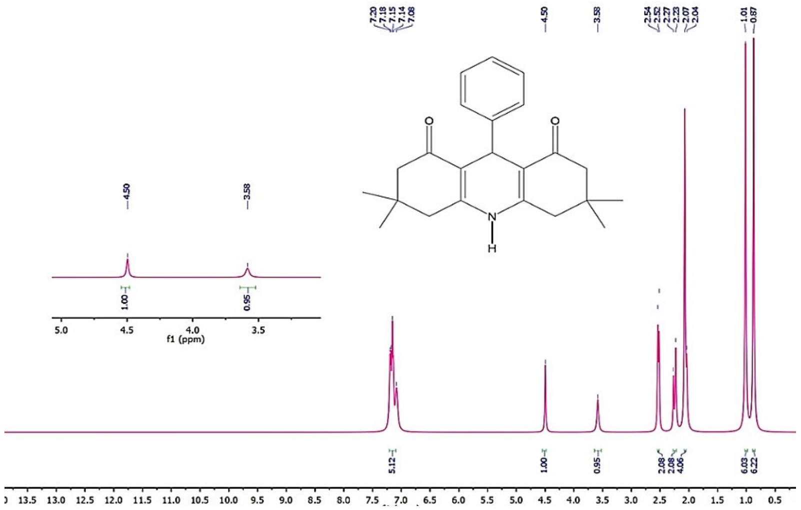

Fig.**4**- ^{1}HNMR spectrum of compound 9-phenyl-3,3,6,6-tetramethyl-3,4,6,7,9,10-hexa-hydro-acridine-1,8-dione

Characterisation of the Products

9-Phenyl-3,3,6,6-Tetramethyl-1,2,3,4,5,6,7,8,-Octahydroxanthene-1,8-dione, White solid. IR (KBr, cm^{-1}): ῡ 2955, 1663, 1364, 1199, 1151, 1005, 698; ^{1}H NMR (DMSO-d6 400 MHz)/ δ (ppm): 0.88 (s, 6H, $2CH_3$), 1.02 (s, 6H, $2CH_3$), 2.04(d, 2H, *J=16 Hz*, CH_2), 2.23 (d, 2H, *J=16 Hz*, CH_2), 2.49 (q, 4H, *J=14 Hz*, $2CH_2$), 4.50 (s, 1H, CH), 7.07-7.22 (m, 5H, ArH)

9- (4-Nitrophenyl) -3,3,6,6-tetramethyl-1,2,3,4,5,6,7,8-octahydroxanthene-1,8-dione, White solid. IR (KBr, cm^{-1}): ῡ 2957, 1663, 1351, 1256, 1202, 1150, 1008, 834; ^{1}H NMR (DMSO-d6 400 MHz)/ δ (ppm): 0.90 (s, 6H, $2CH_3$), 1.04 (s, 6H, $2CH_3$), 2.07 (d, 2H, *J=16 Hz*, CH_2), 2.26 (d, 2H, *J=16 Hz*, CH_2), 2.56 (q, 4H, *J=12 Hz*, $2CH_2$), 4.63 (s, 1H, CH),
7.45 (d, 2H, *J=8 Hz*, ArH), 8.10 (d, 2H, *J=8 Hz*, ArH)

9-(2-chlorophenyl) -3,3,6,6-tetramethyl-3,4,5,6,7,9 -hexahydroxanthen-1,8-dione, light yellow solid. IR (KBr, cm^{-1}): ῡ 2957, 1664, 1359, 1202, 1155, 1033, 752
^{1}H NMR (DMSO-d6 400 MHz)/ δ (ppm): 0.88 (s, 6H, $2CH_3$), 1.01 (s, 6H, $2CH_3$), 1.99-2.48 (m, 8H, $4CH_2$), 4.79 (s, 1H, CH), 7.09-7.22 (m, 4H, ArH)

9-(3- Nitrophenyl) -3,3,6,6-tetramethyl-1,2,3,4,5,6,7,8-hexahydroxanthen-1,8-dione, White solid.

IR (KBr, cm-1): ῡ 2956, 1665, 1357, 1198, 1147, 1004, 570; 1H NMR (DMSO-d6 400 MHz)/δ (ppm): 0.90 (s, 6H, 2CH3), 1.04 (s, 6H, 2CH3), 2.08 (d, 2H, J=16 Hz, CH2), 2.27 (d, 2H, J=16 Hz , CH2), 2.59 (q, 4H, 2CH2), 4.64 (s, 1H, CH), 7.56-7.66 (m, 3H, ArH), 8.00, (s, 1H, ArH)

9-(4-Bromo) -3,3,6,6-tetramethyl-1,2,3,4,5,6,7,8-hexahydroxanthen-1,8-dione, White solid. IR (KBr, cm^{-1}): ῡ 2952, 1662, 1363, 1197, 1152, 1007, 843; 1H NMR (DMSO-d6 400 MHz)/δ (ppm): 0.90 (s, 6H, 2CH3), 1.03 (s, 6H, 2CH3), 2.10-2.27 (m, 8H, 4CH2), 4.48 (s, 1H, CH), 7.13-7.42 (m, 4H, ArH)

9-(4-Methoxyphenyl)-3,3,6,6-tetramethyl-1,2,3,4,5,6,7,8-hexahydroxanthen-1,8-dione, White solid. IR (KBr, cm^{-1}): ῡ 2951, 1667, 1359, 1256, 1192, 1031, 824; 1H NMR (DMSO-d6 400 MHz)/δ (ppm): 0.90 (s, 6H, 2CH3), 1.04 (s, 6H, 2CH3), 2.08-2.26 (m, 8H, 4CH2), 3.68 (s, 3H, OMe), 4.64 (s, 1H, CH), 6.77-67.08 (m, 4H, ArH)

9-(4-Fluorophenyl)-3,3,6,6-tetramethyl-1,2,3,4,5,6,7,8-hexahydroxanthen-1,8-dione, White solid.
IR (KBr, cm^{-1}): ῡ 2955, 1663, 1365, 1201, 1153, 1150, 1006, 788; ^{1}H NMR (DMSO-d6 400 MHz)/ δ (ppm): 0.90 (s, 6H, 2CH_3), 1.03 (s, 6H, 2CH_3), 2.06-2.55 (m, 8H, 4CH_2), 4.51 (s, 1H, CH), 7.02-7.19 (m, 4H, ArH)

4- (3,3,6,6-Tetramethyl-1,8-dioxo-2,3,4,5,6,7,8,9-octahydro-xanthen-9-yl) benzaldehyde, White solid. IR (KBr, cm^{-1}): ῡ 2954, 1667, 1366, 1200, 1155, 1008, 814; 1H NMR (DMSO-d6 400 MHz)/δ (ppm): 0.89 (s, 6H, 2CH3), 1.04 (s, 6H, 2CH3), 2.09-2.26 (m, 8H, 4CH2), 4.59 (s, 1H, CH), 7.41-7.78 (m, 4H, ArH), 9.90 (s, 1H, COH)

9-(4-Bromo) -3,3,6,6 -tetramethyl-3,4,6,7,9,10, -hexahydroacridine-1,8-dione, Yellow solid. IR (KBr, cm^{-1}): ῡ 2953, 1663, 1363, 1197, 1153, 1007, 843; ^{1}H NMR (DMSO-d6 400 MHz)/ δ (ppm): 0.88 (s, 6H, CH_3), 1.02 (s, 6H, CH_3), 2.05 (d, 2H, *J=16 Hz*, CH_2), 2.23 (d, 2H, *J=16 Hz*, CH_2), 2.52 (s, 4H, 2CH_2), 3.59 (s. 1H, NH), 4.47 (s, 1H, CH), 7.10 (d, 2H, *J=8 Hz*, ArH), 7.39 (d, 2H, *J=8 Hz*, ArH)

9-Phenyl-3,3,6,6-tetramethyl-3,4,6,7,9,10- hexahydroacridine-1,8-dione, Yellow solid. IR (KBr, cm^{-1}): ῡ .2954, 1336, 1664, 1199, 1152, 1007, 801; ^{1}H NMR (DMSO-d6 400 MHz)/ δ (ppm): 0.87 (s, 6H, 2CH_3), 1.01 (s, 6H, 2CH_3), 2.04 (q, 4H, *J=12 Hz*, 2CH_2), 2.23 (d, 2H, *J=16 Hz*, CH_2), 2.52 (d, 2H, *J=8 Hz*, CH_2), 3.58 (s. 1H, NH), 4.50 (s, 1H, CH), 7.08-720 (m, 5H, ArH)

9-(2-Chlorophenyl)-3,3,6,6 -tetramethyl-3,4,6,7,9,10- hexahydroacridine-1,8-dione, White solid. IR (KBr, cm^{-1}): ῡ 2957, 1666, 1357, 1203, 1156, 1009, 750; ^{1}H NMR (DMSO-d6 400 MHz)/ δ (ppm): 0.90 (s, 6H, 2CH_3), 1.03 (s, 6H, 2CH_3), 2.01 (d, 2H, *J=16 Hz*, CH_2), 2.22 (d, 2H, *J=16 Hz*, CH_2), 2.43-2.60 (m, 4H, 2CH_2), 3.59 (s. 1H, NH), 4.81 (s, 1H, CH), 7.11, (d, 2H, , *J=8 Hz*, ArH), 7.20-7.26 (m, 2H, ArH)

9-(3-Nitrophenyl)-3,3,6,6- tetramethyl-3,4,6,7,9,10 -hexahydroacridine-1,8-dione, White solid.

IR (KBr, cm^{-1}): ῡ 2957, 1664, 1357, 1199, 1147, 1004, 922; ^{1}H NMR (DMSO-d6 400 MHz)/ δ (ppm): 0.86 (s, 6H, $2CH_3$), 1.00 (s, 6H, $2CH_3$), 2.04 (d, 2H, *J=16 Hz*, CH_2), 2.23 (d, 2H, *J=12 Hz*, CH_2), 2.47-2.58 (m, 4H, $2CH_2$), 3.29 (s. 1H, NH), 4.60 (s, 1H, CH), 7.49-7.63 (m, 3H, ArH), 7.96 (s, 1H, ArH)

9-(4-Methoxyphenyl)-3,3,6,6-tetramethyl-3,4,6,7,9,10- hexahydroacridine-1,8-dione, White solid.

IR (KBr, cm^{-1}): ῡ 2952, 1667, 1359, 1257, 1192, 1031, 842; 1H NMR (DMSO-d6 400 MHz)/δ (ppm): 0.88 (s, 6H, 2CH3), 1.01 (s, 6H, 2CH3), 1.74-2.23 (m, 8H, 4CH2), 3.58 (s. 1H, NH), 3.65 (s, 1H, OMe), 4.43 (s, 1H, CH), 6.75-7.04 (m, 4H, ArH)

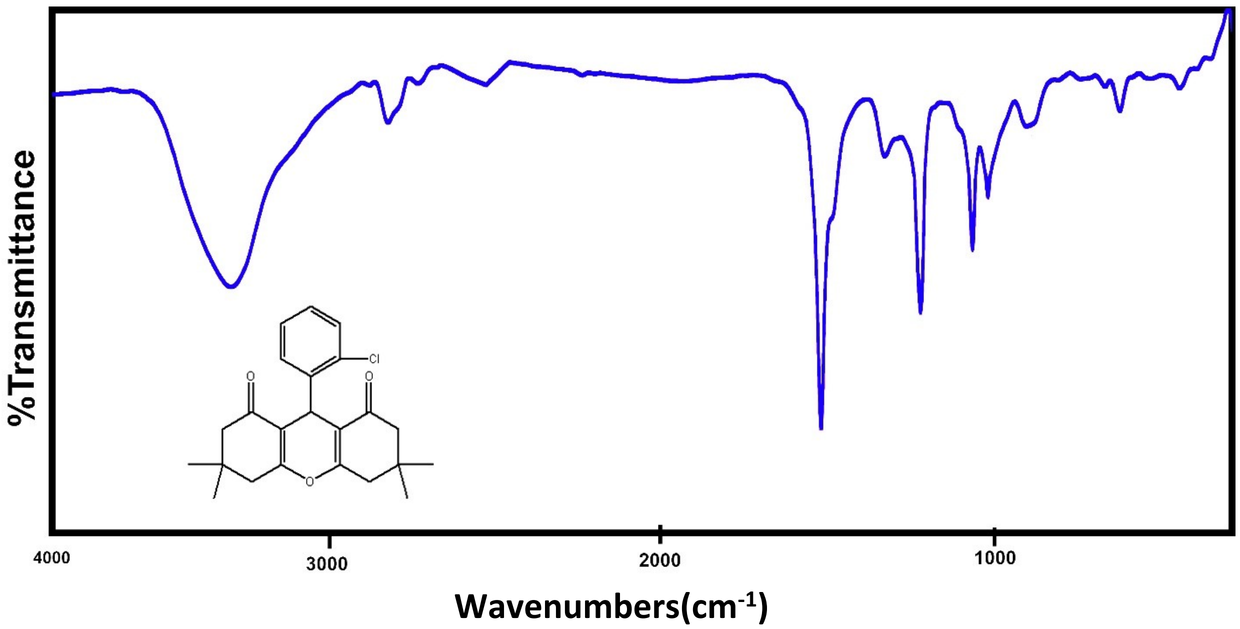

Fig.5- Infrared products of 9-(2-chlorophenyl)-3,3,6,6-tetramethyl-1,2,3,4,5,6,7,8-octa-hydro-xanthenes -1,8-dione

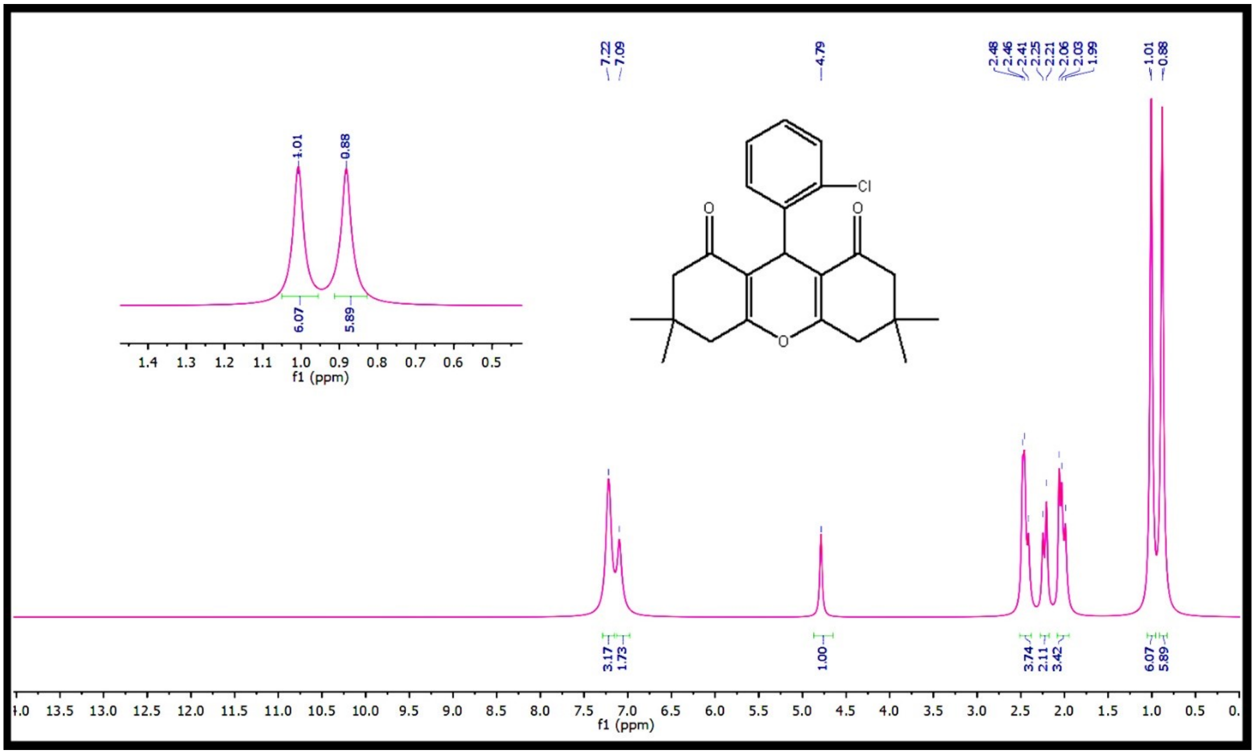

Fig.6- ^{1}HNMR products of 9-(2-chlorophenyl)-3,3,6,6-tetramethyl-1,2,3,4,5,6,7,8-octa-hydro-xanthenes -1,8-dione

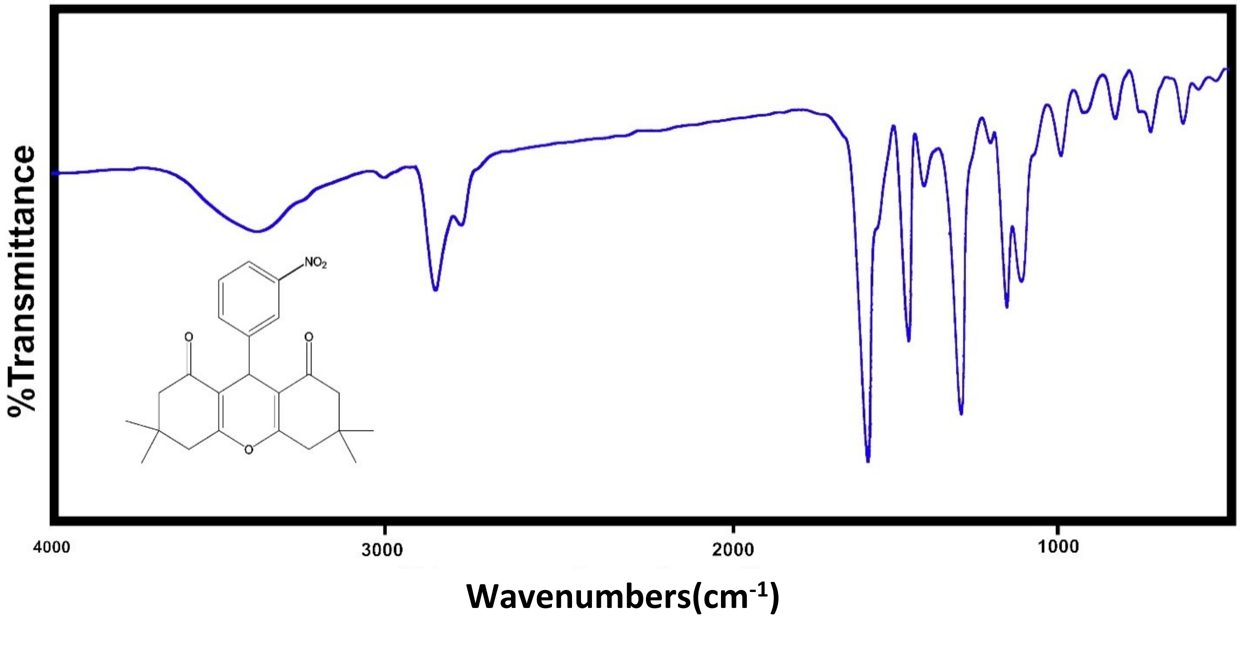

Fig.7- Infrared products of 9-(3-nitrophenyl)-3,3,6,6-tetramethyl-1,2,3,4,5,6,7,8-octa-hydro-xanthenes -1,8-dione

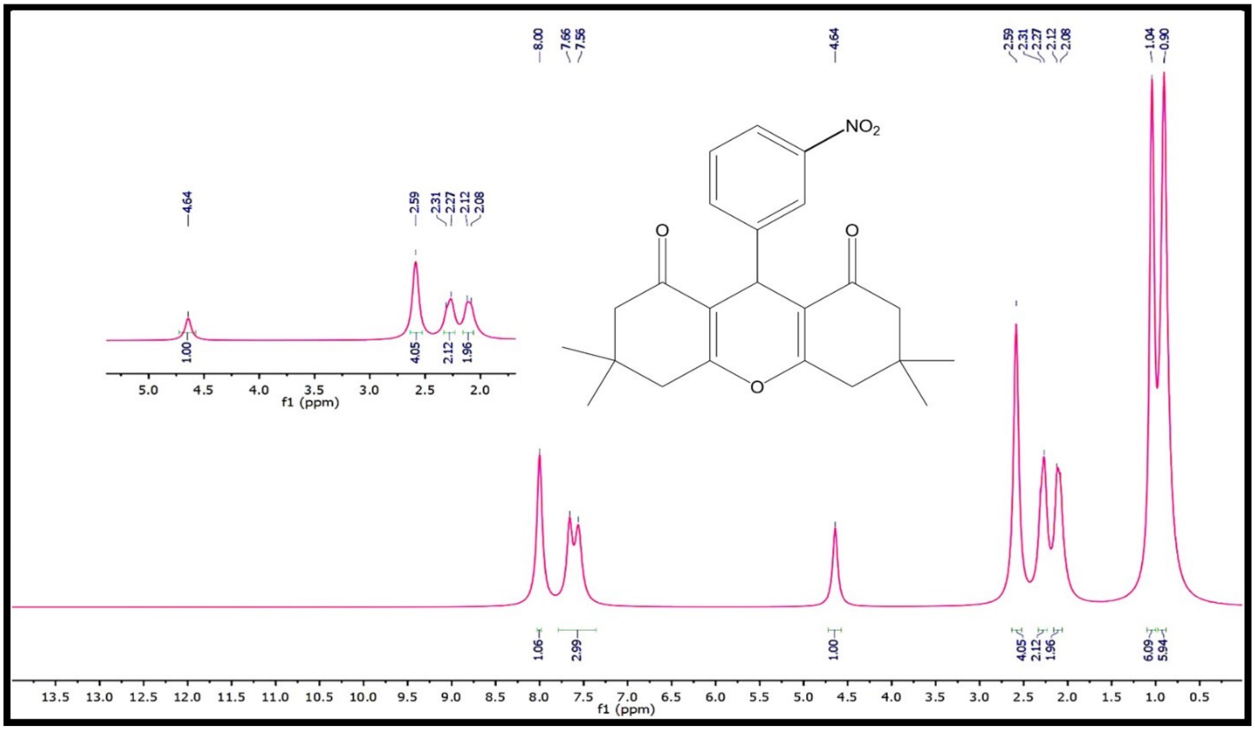

Fig.8- ^{1}HNMR products of 9-(3-nitrophenyl)-3,3,6,6-tetramethyl-1,2,3,4,5,6,7,8-octa-hydro-xanthenes -1,8-dione

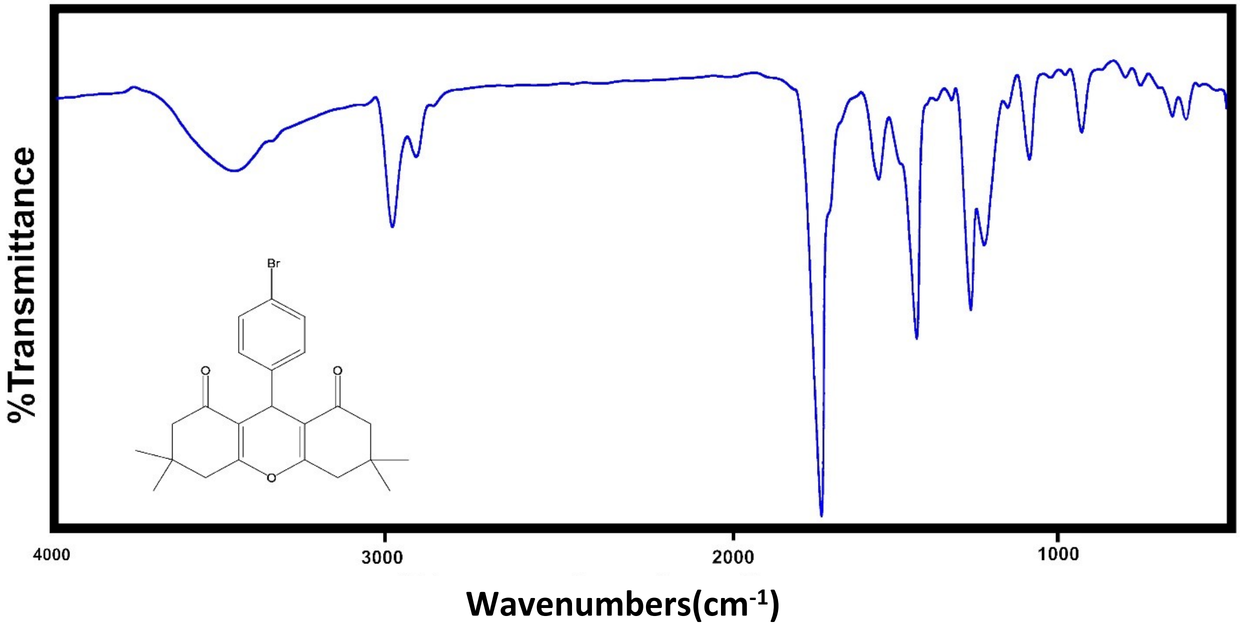

Fig.9- Infrared products of 9-(4-bromophenyl)-3,3,6,6-tetramethyl-1,2,3,4,5,6,7,8-octa-hydro-xanthenes -1,8-dione

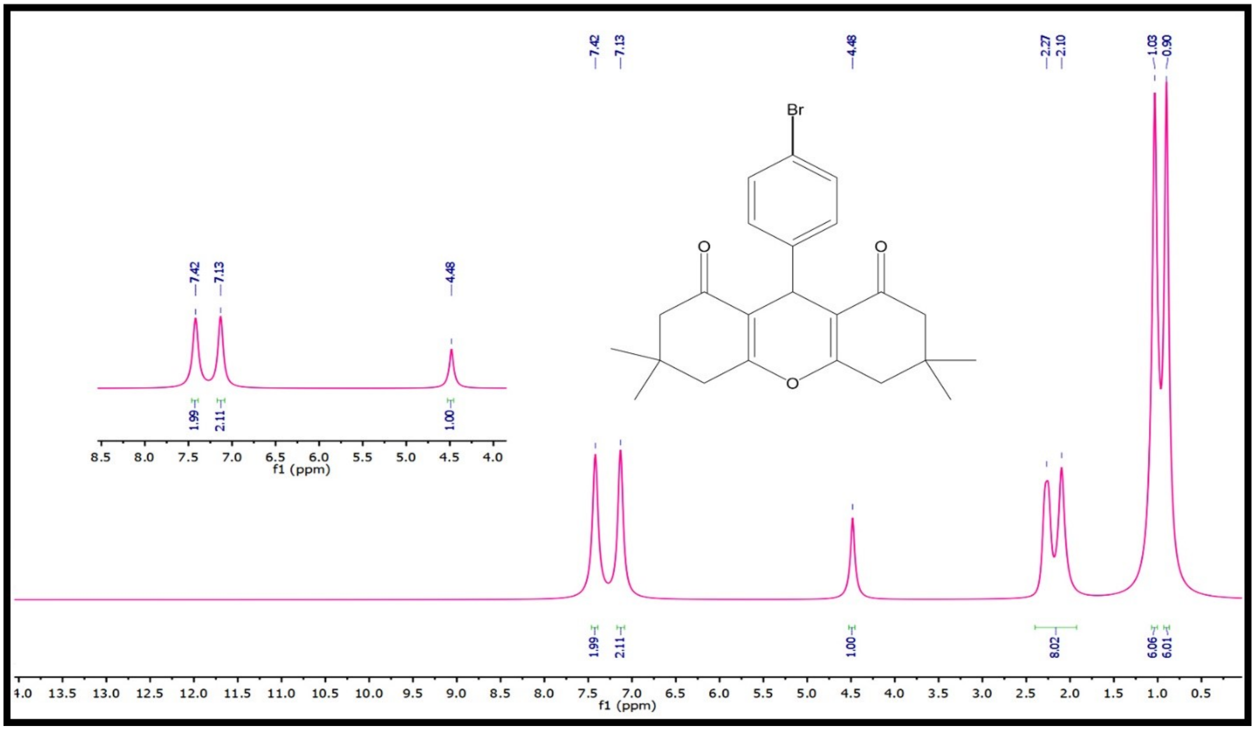

Fig.10- ^{1}HNMR products of 9-(4-bromophenyl)-3,3,6,6-tetramethyl-1,2,3,4,5,6,7,8-octa-hydro-xanthenes -1,8-dione

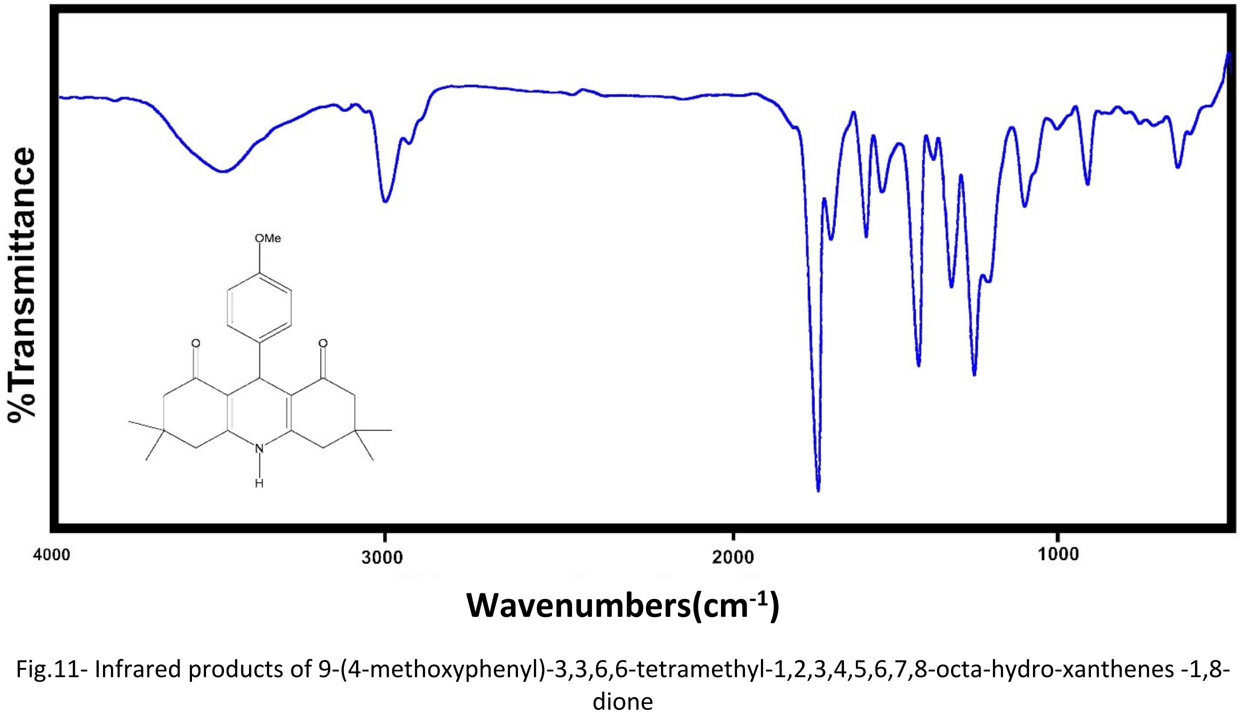

Fig.11- Infrared products of 9-(4-methoxyphenyl)-3,3,6,6-tetramethyl-1,2,3,4,5,6,7,8-octa-hydro-xanthenes -1,8-dione

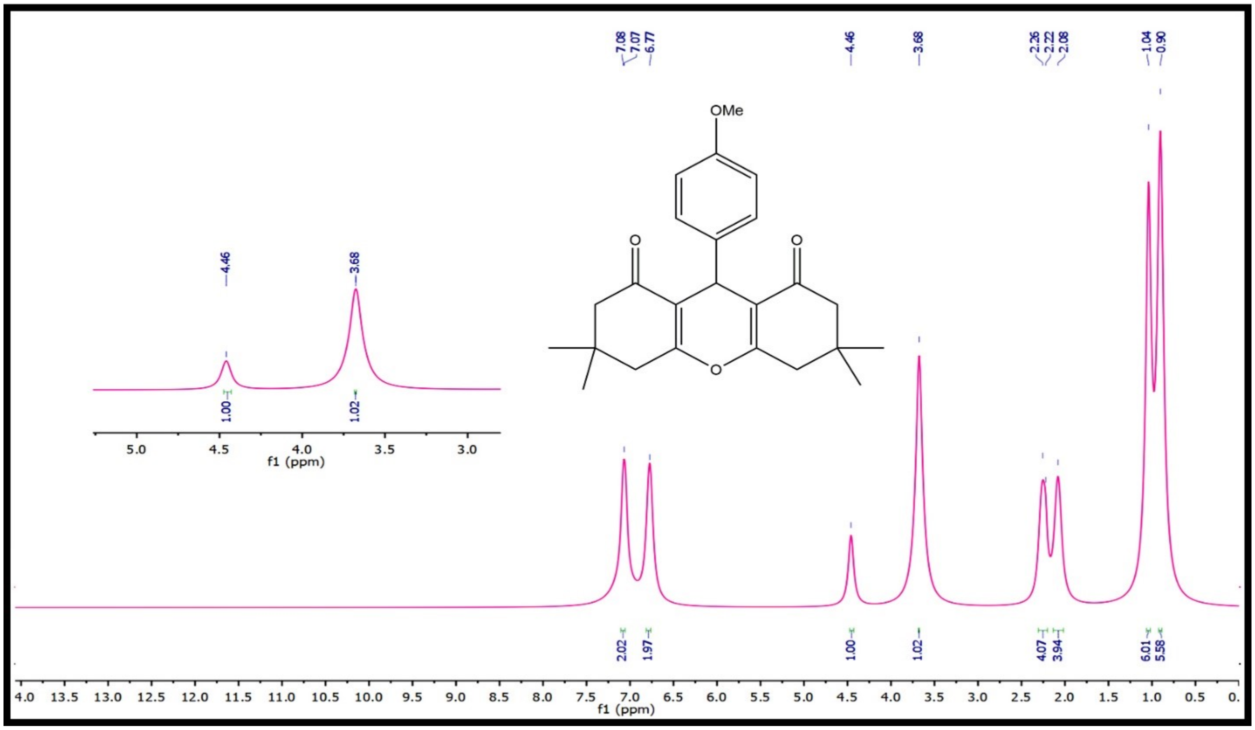

Fig.12- ^{1}HNMR products of 9-(4-methoxyphenyl)-3,3,6,6-tetramethyl-1,2,3,4,5,6,7,8-octa-hydro-xanthenes -1,8-dione

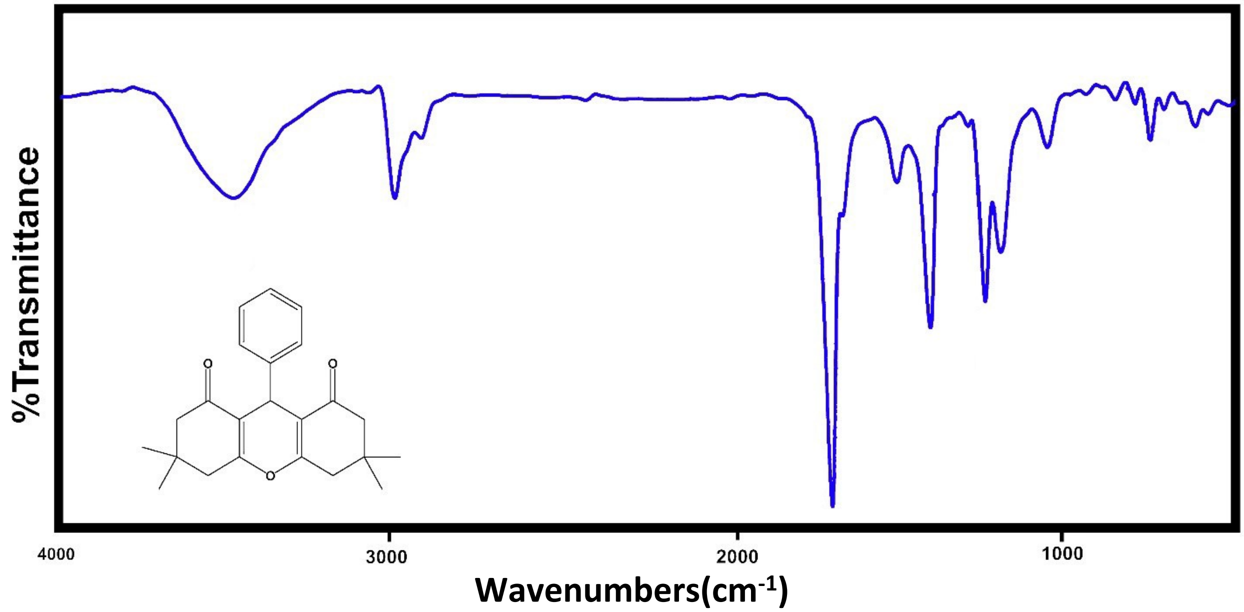

Fig.13- Infrared products of 9-phenyl-3,3,6,6-tetramethyl-1,2,3,4,5,6,7,8-octa-hydro-xanthenes -1,8-dione

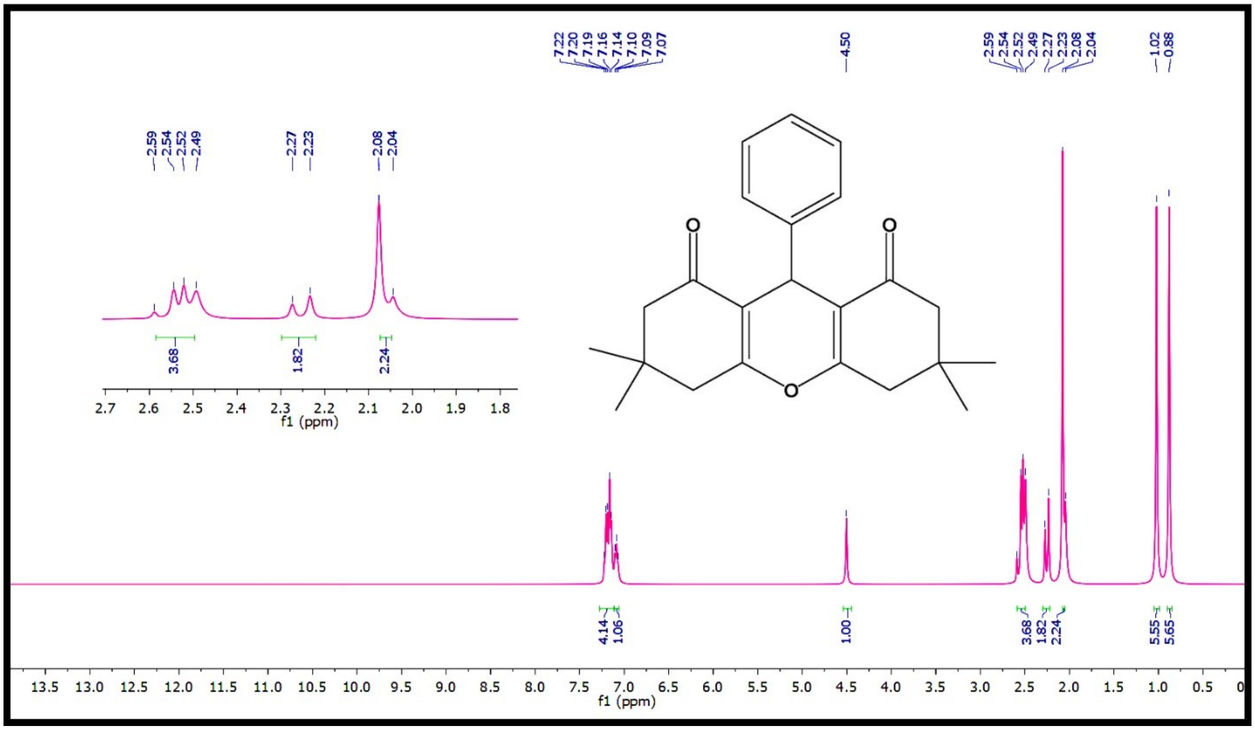

Fig.14- ^{1}HNMR products of 9-phenyl-3,3,6,6-tetramethyl-1,2,3,4,5,6,7,8-octa-hydro-xanthenes -1,8-dione

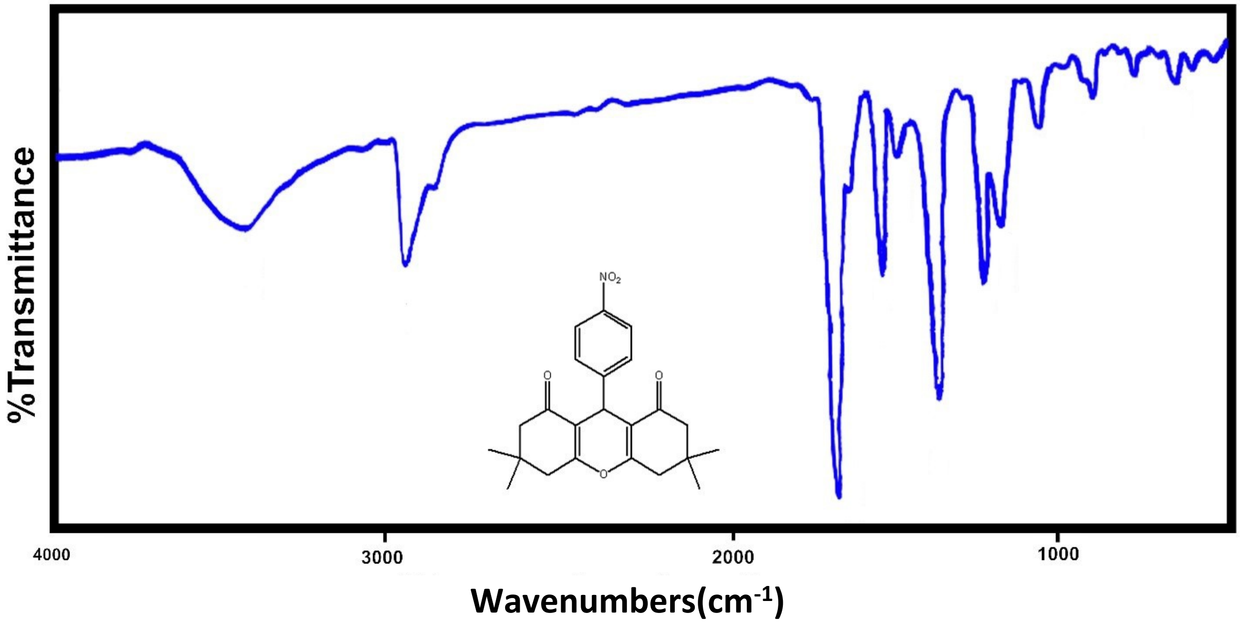

Fig.15- Infrared products of 9-(4-nitrophenyl)-3,3,6,6-tetramethyl-1,2,3,4,5,6,7,8-octa-hydro-xanthenes -1,8-dione

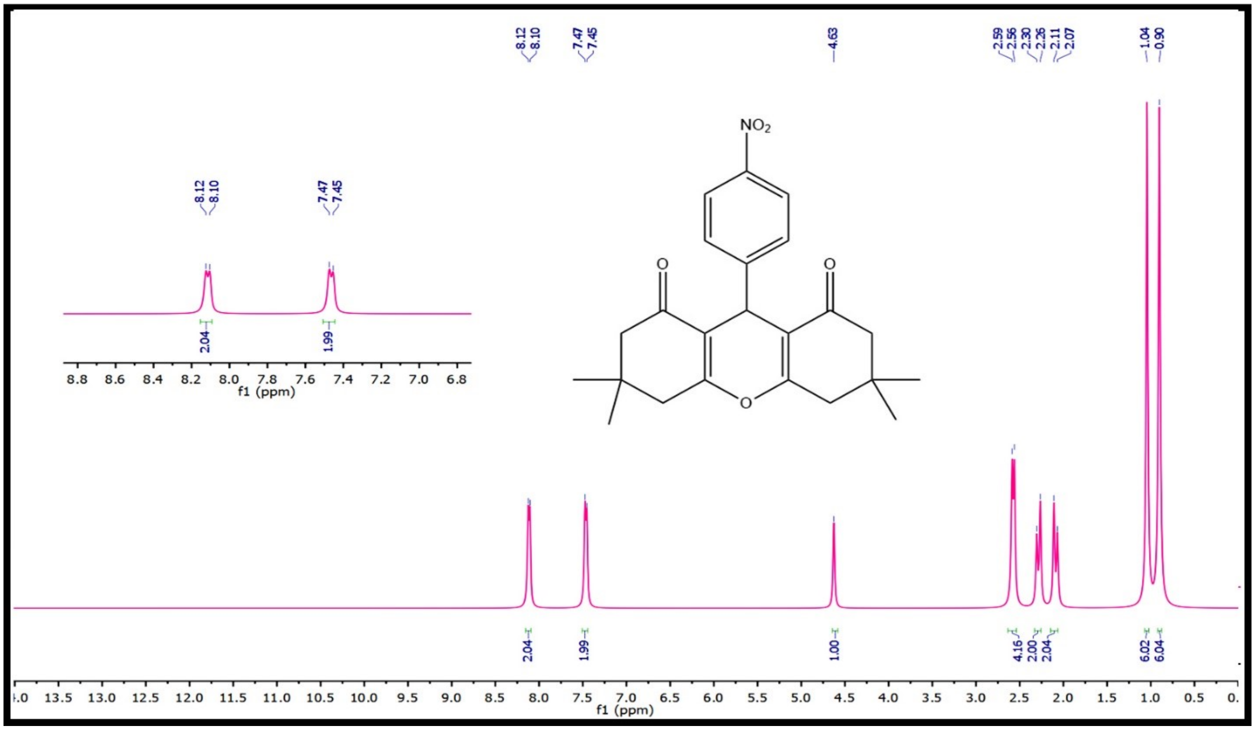

Fig.16- ^{1}HNMR products of 9-(4-nitrophenyl)-3,3,6,6-tetramethyl-1,2,3,4,5,6,7,8-octa-hydro-xanthenes -1,8-dione

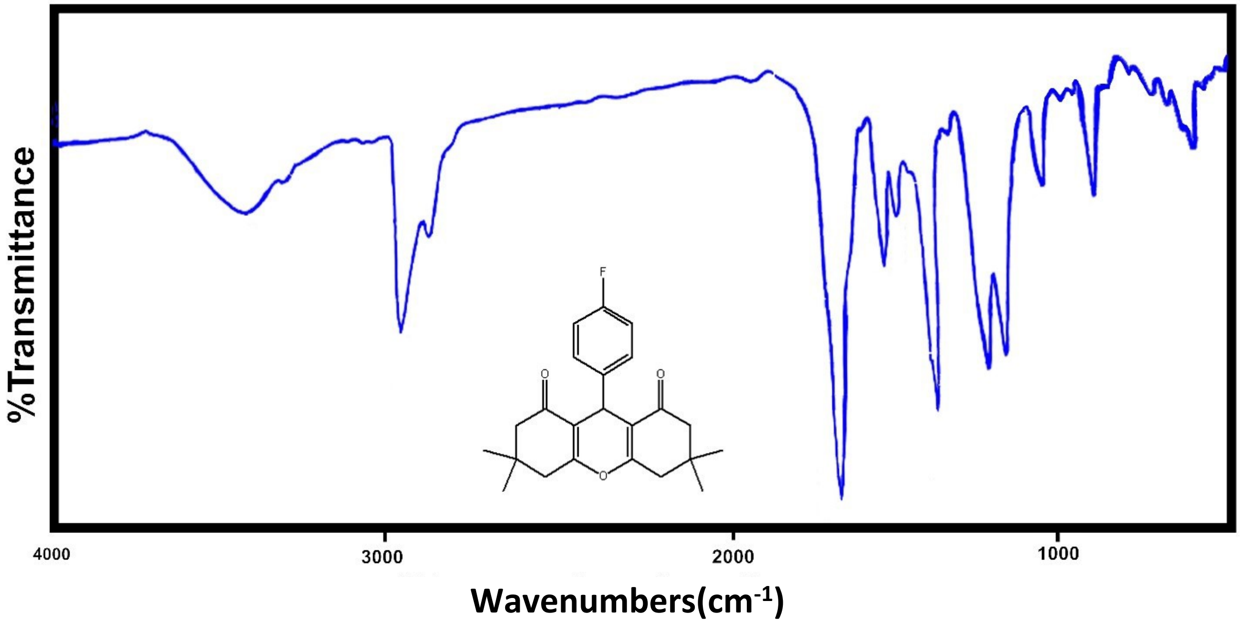

Fig.17- Infrared products of 9-(4- fluorophenyl)-3,3,6,6-tetramethyl-1,2,3,4,5,6,7,8-octa-hydro-xanthenes -1,8-dione

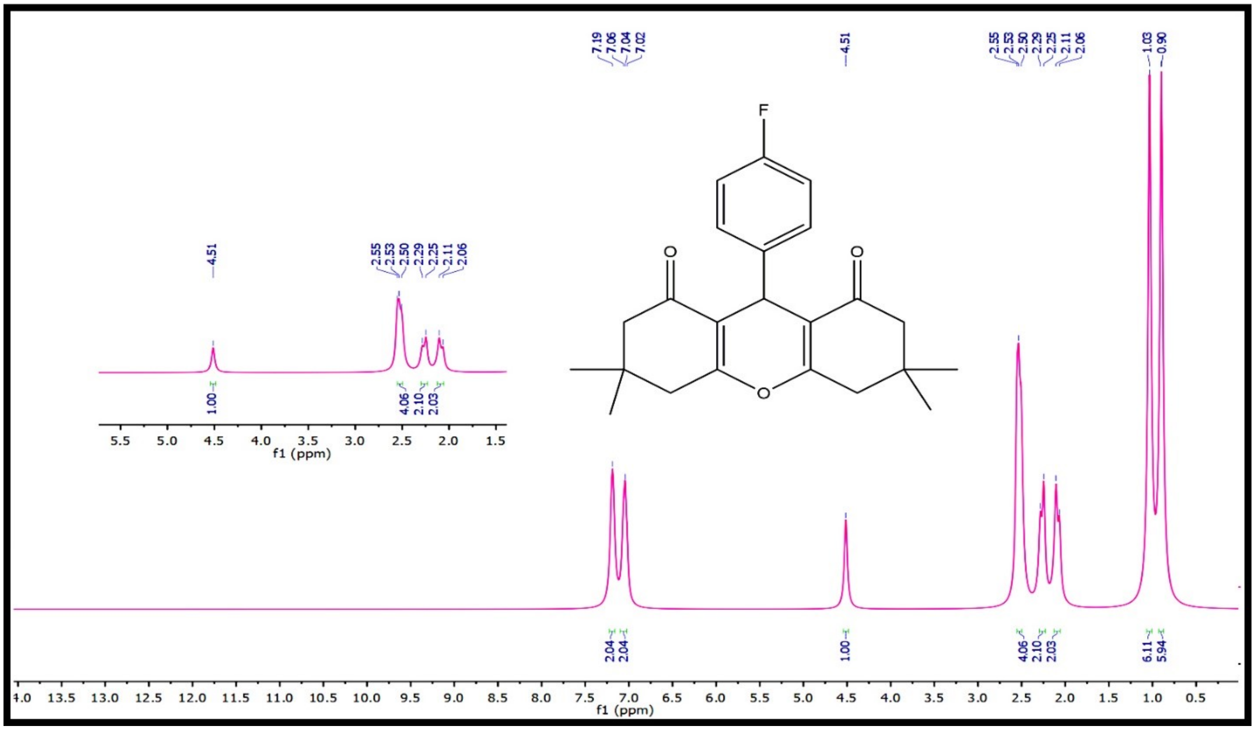

Fig.18- ^{1}HNMR products of 9-(4-fluorophenyl)-3,3,6,6-tetramethyl-1,2,3,4,5,6,7,8-octa-hydro-xanthenes -1,8-dione

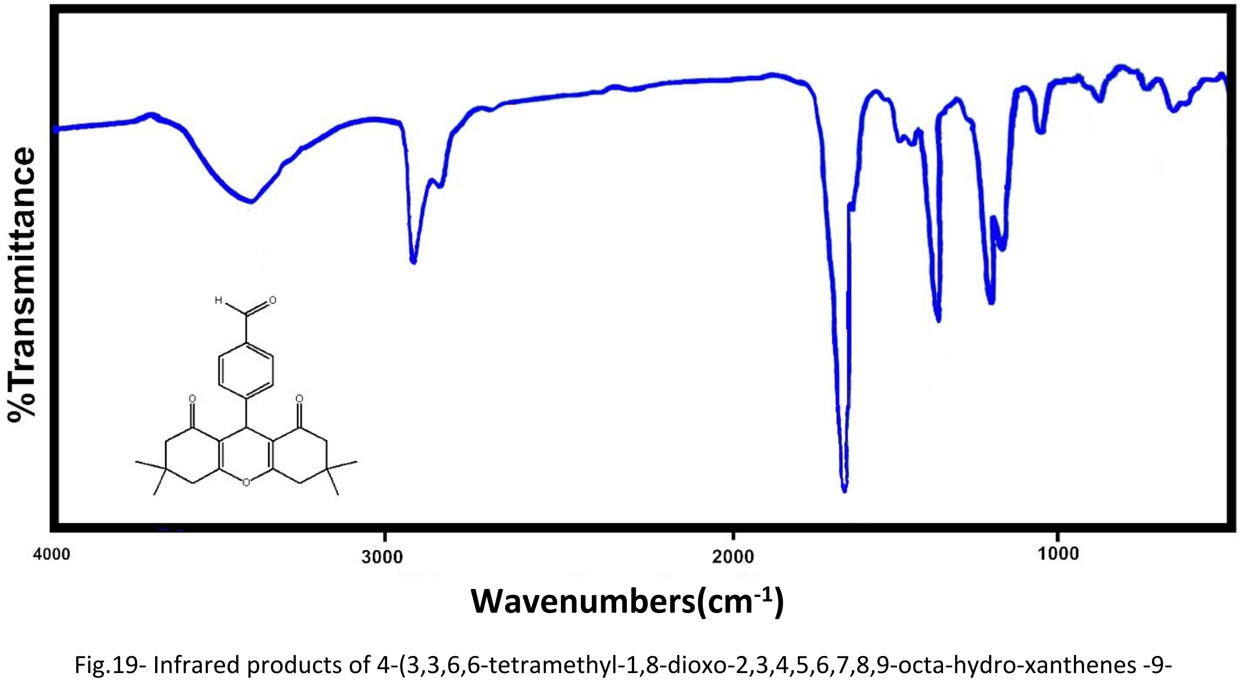

Fig.19- Infrared products of 4-(3,3,6,6-tetramethyl-1,8-dioxo-2,3,4,5,6,7,8,9-octa-hydro-xanthenes -9-iyl)benzaldehyde

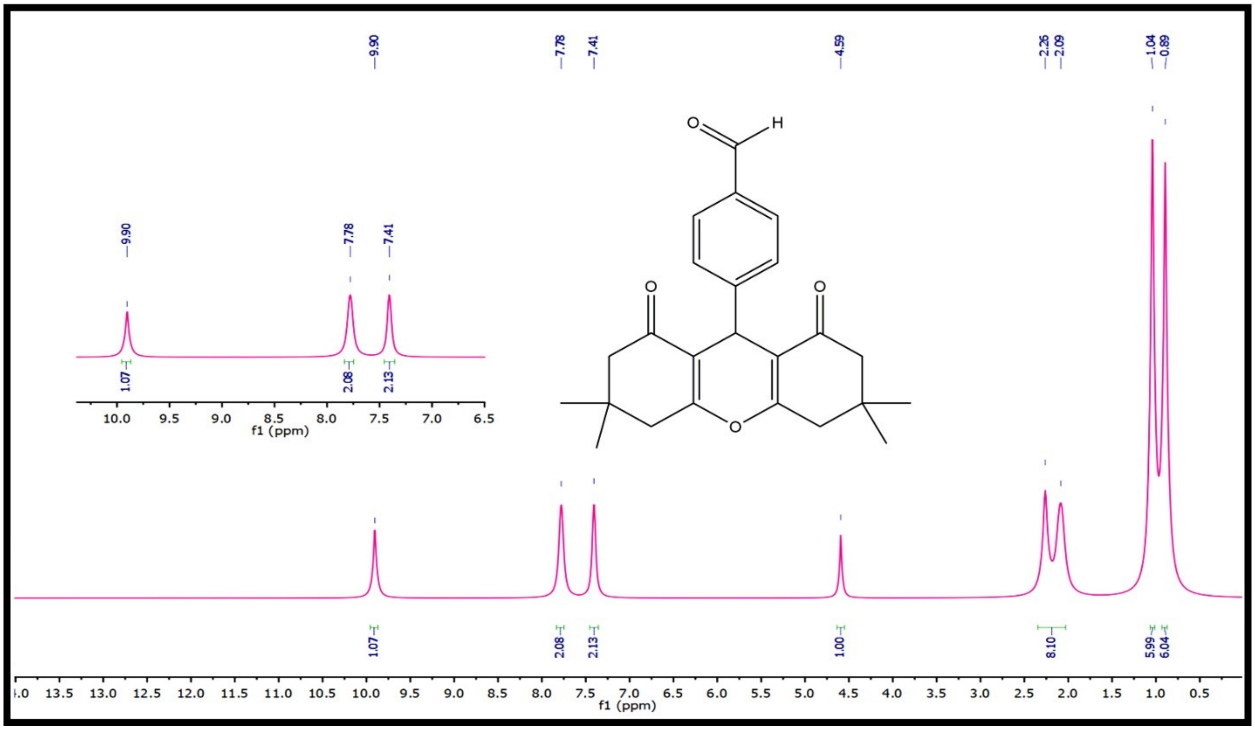

Fig.20- ^{1}HNMR products of 4-(3,3,6,6-tetramethyl-1,8-dioxo-2,3,4,5,6,7,8,9-octa-hydro-xanthenes -9-iyl)benzaldehyde

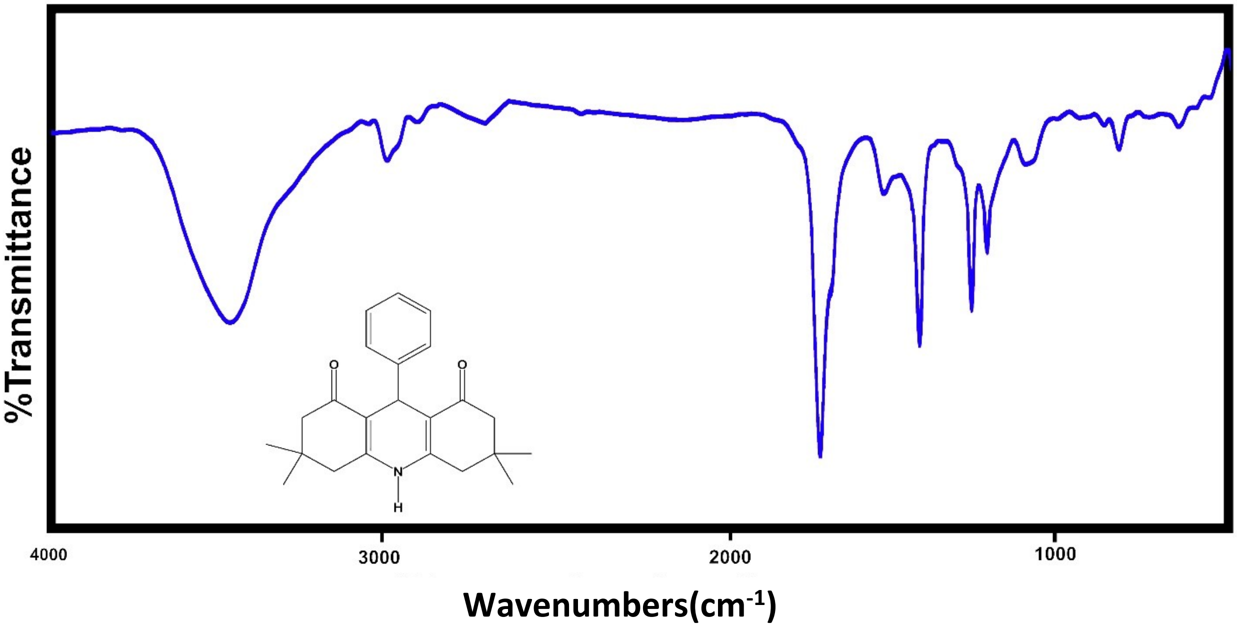

Fig.21- Infrared products of 9-phenyl-3,3,6,6-tetramethyl-3,4,6,7,9,10-hexa-hydro-acridine -1,8-dione

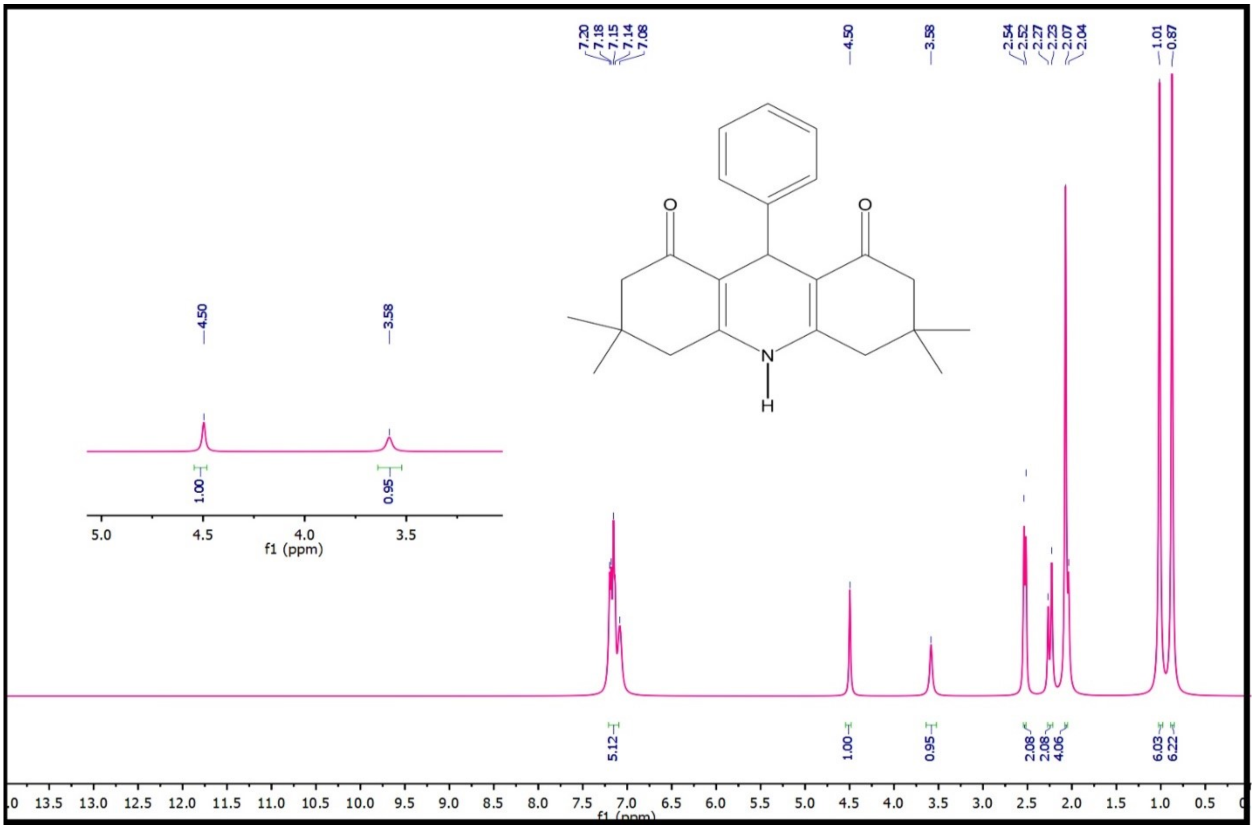

Fig.22- 1HNMR products of 9-phenyl-3,3,6,6-tetramethyl-3,4,6,7,9,10-hexa-hydro-acridine -1,8-dione

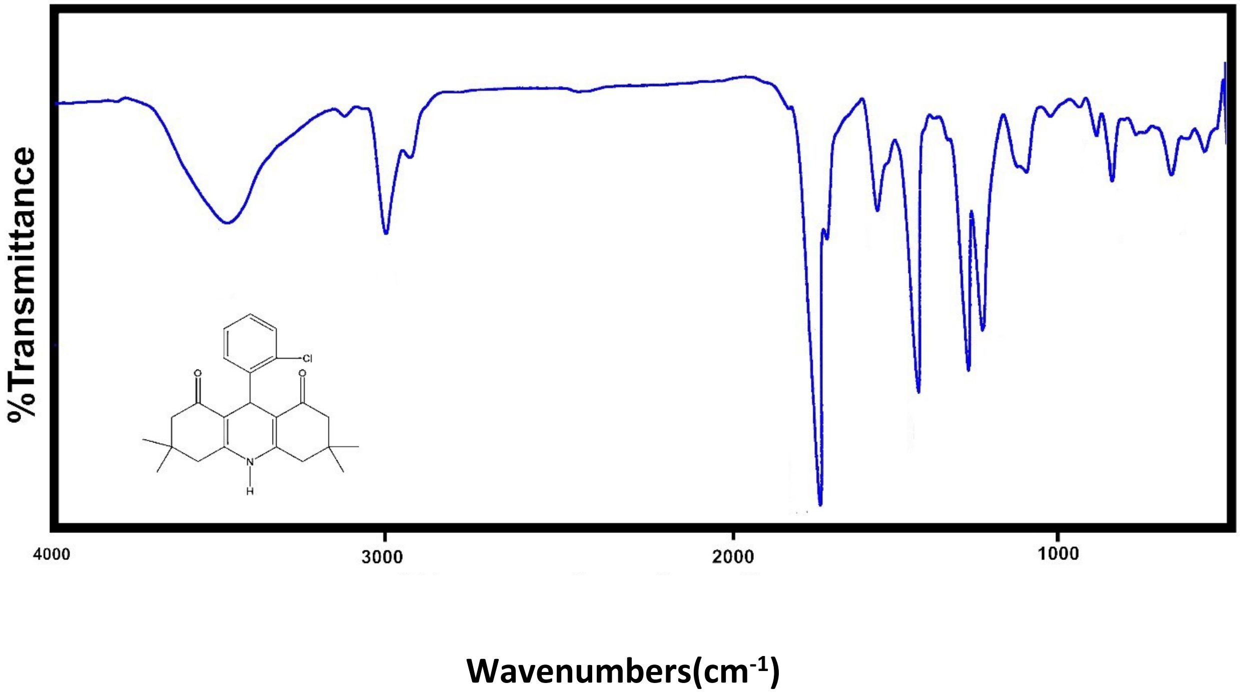

Fig.23- Infrared products of 9-(2-chlorophenyl)-3,3,6,6-tetramethyl-3,4,6,7,9,10-hexa-hydro-acridine -1,8-dione

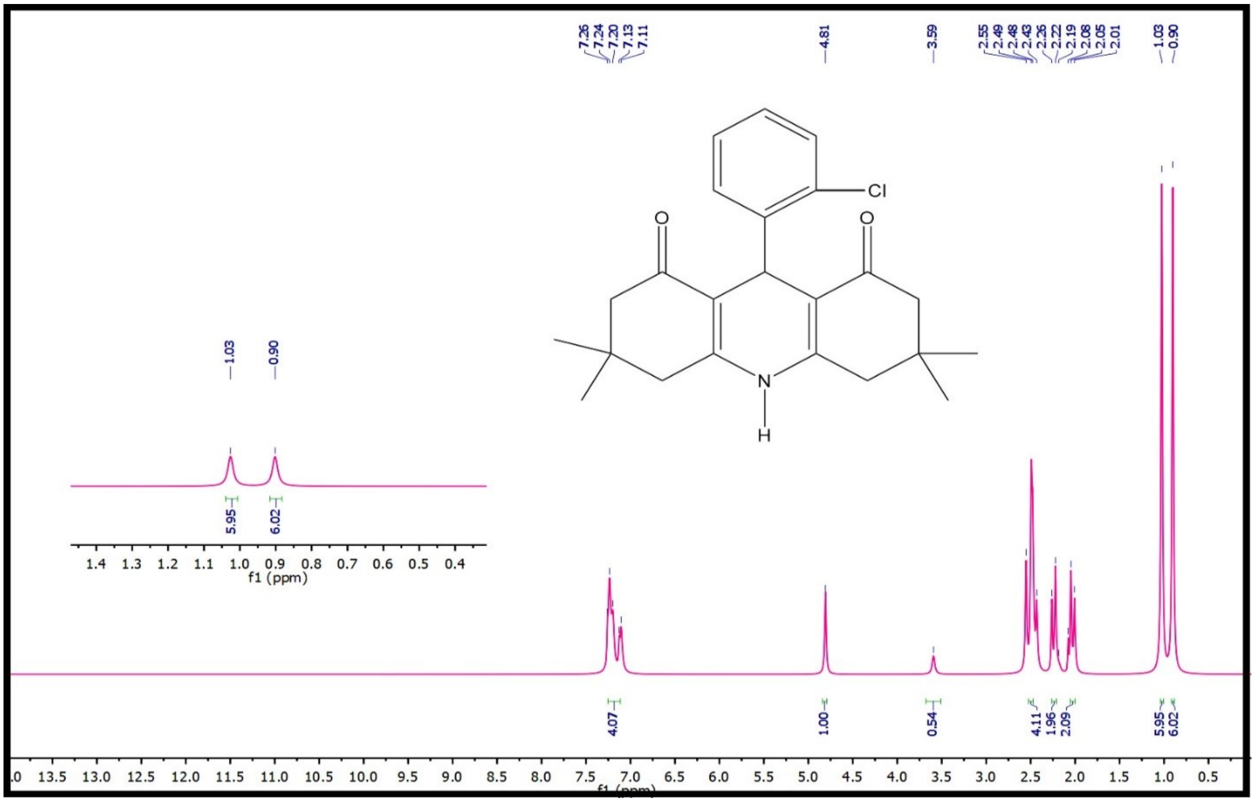

Fig.24- ^{1}HNMR products of 9-(2-chlorophenyl)-3,3,6,6-tetramethyl-3,4,6,7,9,10-hexa-hydro-acridine -1,8-dione

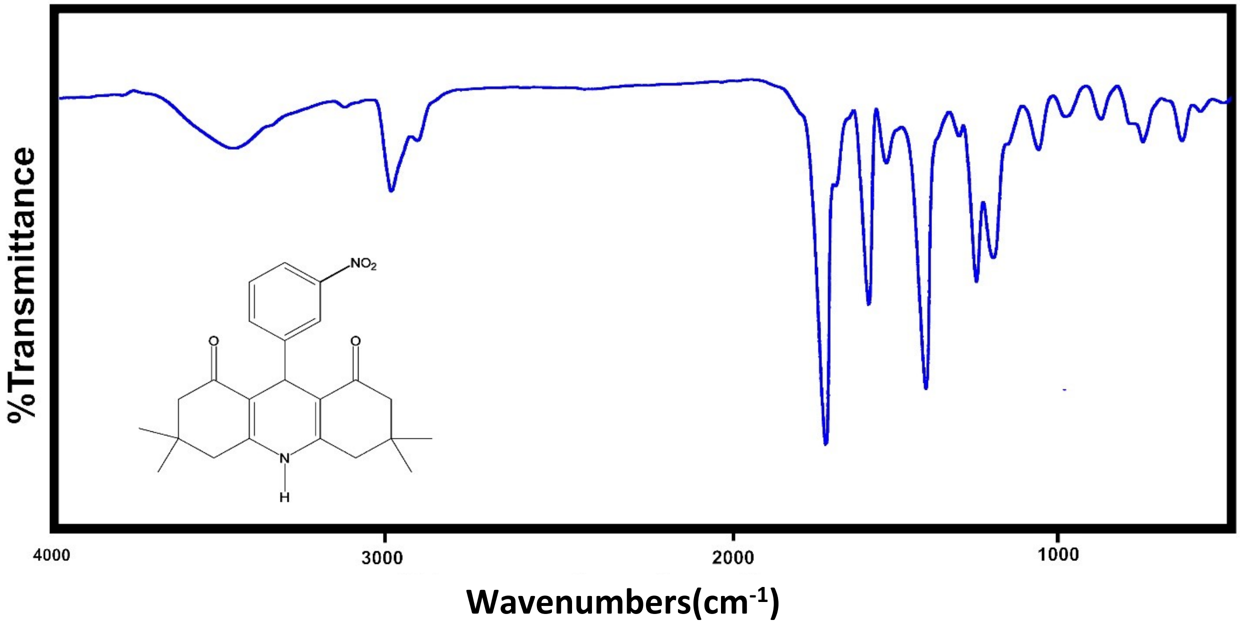

Fig.25- Infrared products of 9-(3-nitrophenyl)-3,3,6,6-tetramethyl-3,4,6,7,9,10-hexa-hydro-acridine -1,8-dione

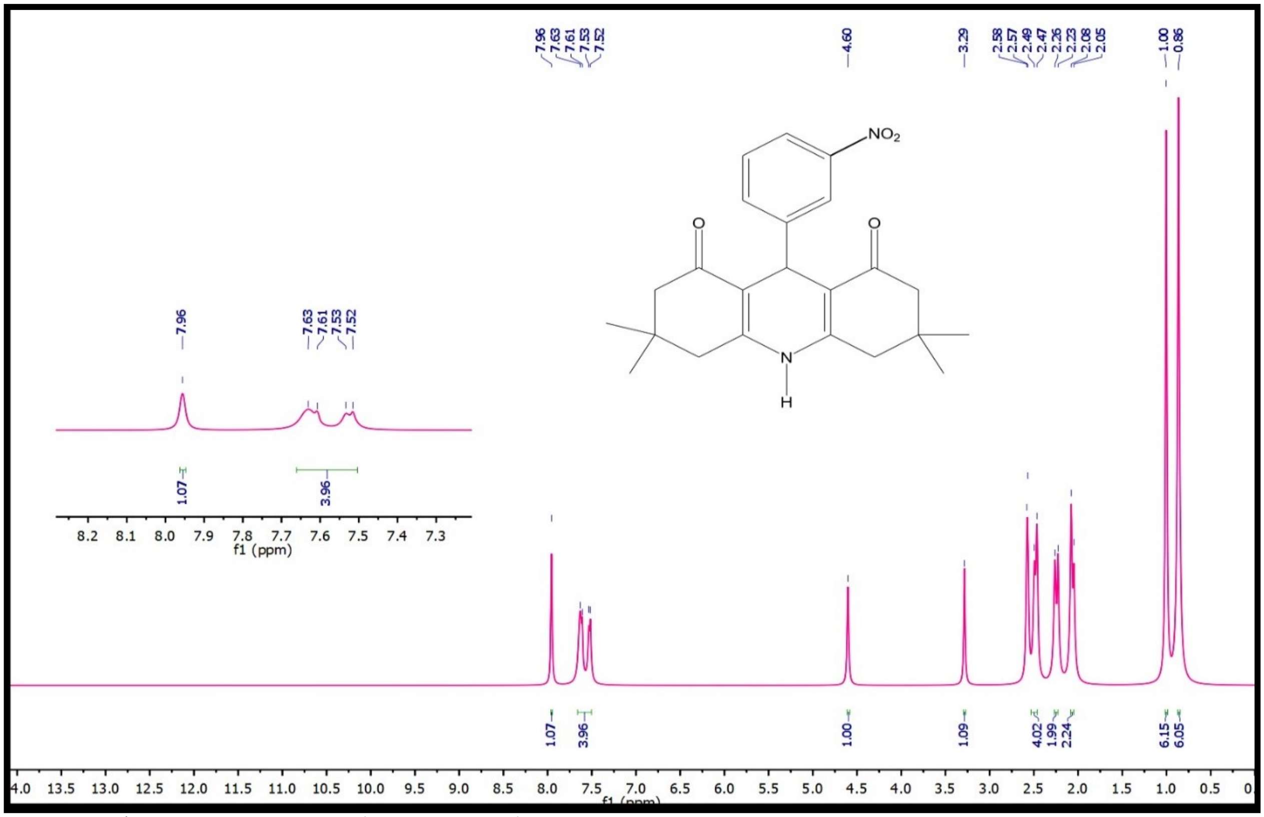

Fig.26- ^{1}HNMR products of 9-(3-nitrophenyl)-3,3,6,6-tetramethyl-3,4,6,7,9,10-hexa-hydro-acridine -1,8-dione

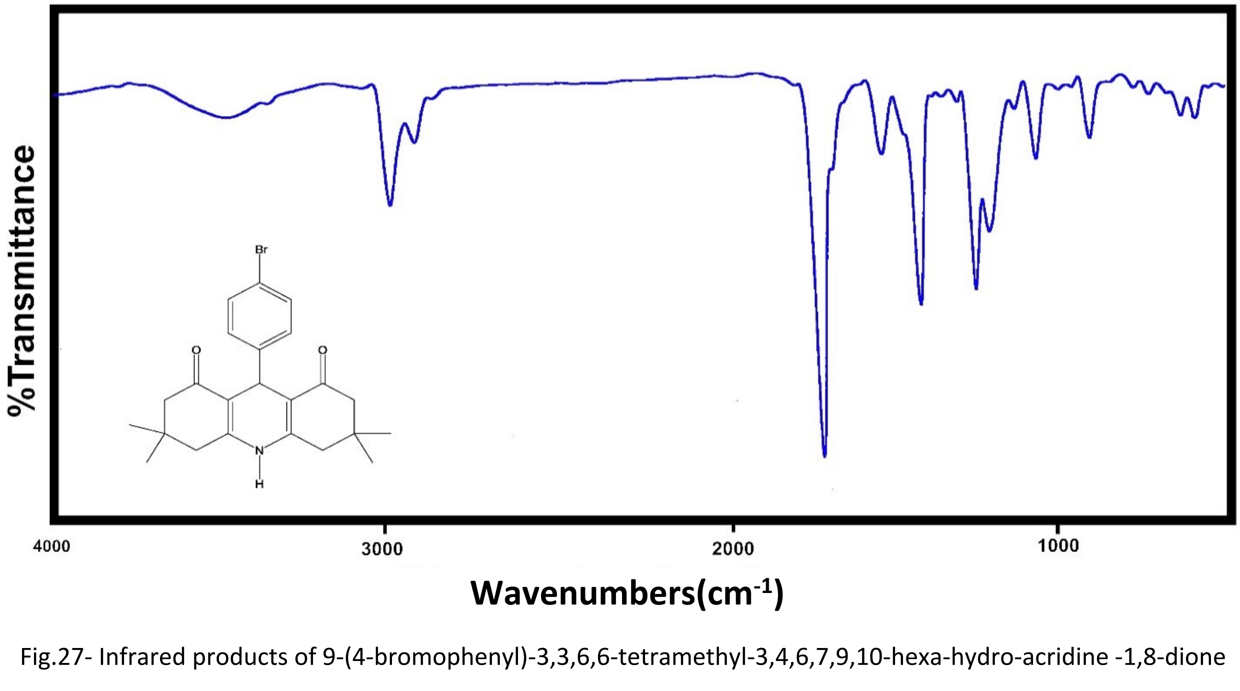

Fig.27- Infrared products of 9-(4-bromophenyl)-3,3,6,6-tetramethyl-3,4,6,7,9,10-hexa-hydro-acridine -1,8-dione

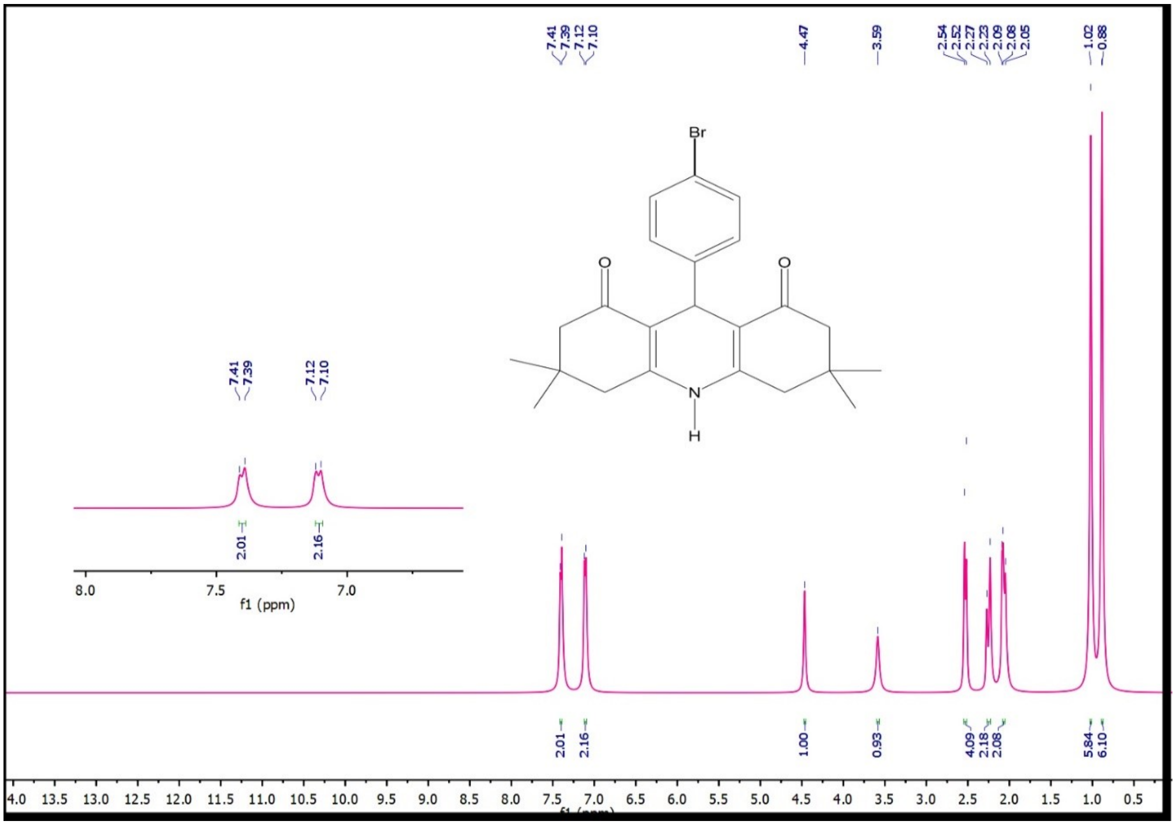

Fig.28- ^{1}HNMR products of 9-(4-bromophenyl)-3,3,6,6-tetramethyl-3,4,6,7,9,10-hexa-hydro-acridine -1,8-dione

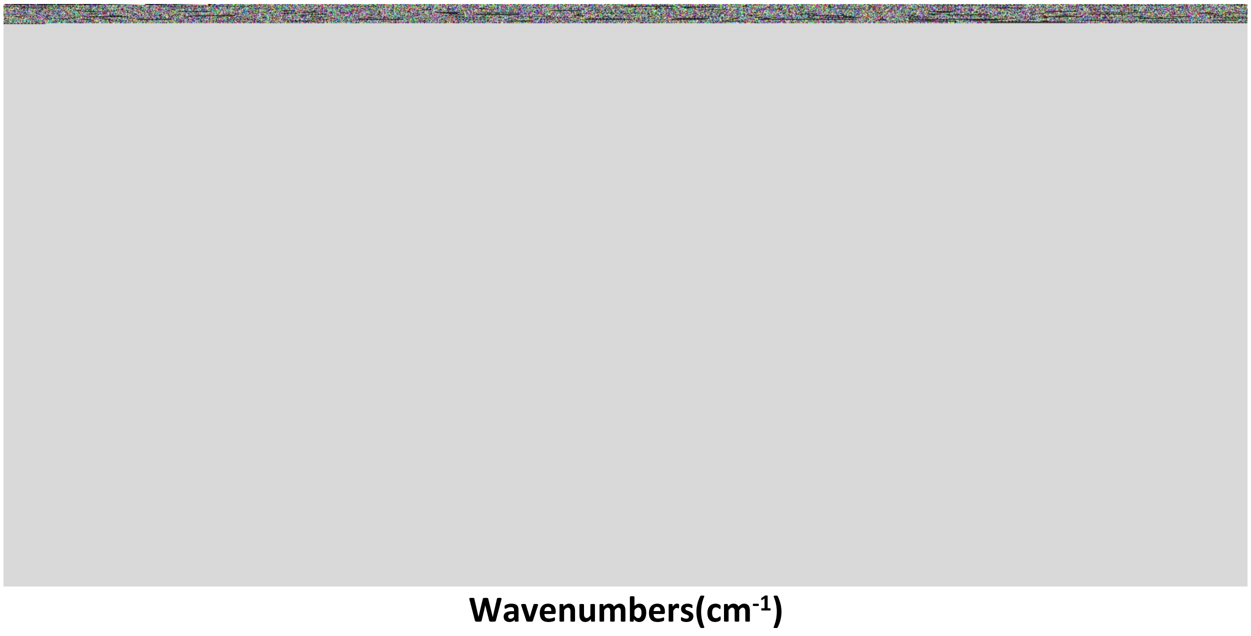

Fig.29- Infrared products of 9-(4-methoxyphenyl)-3,3,6,6-tetramethyl-3,4,6,7,9,10-hexa-hydro-acridine -1,8-dione

-

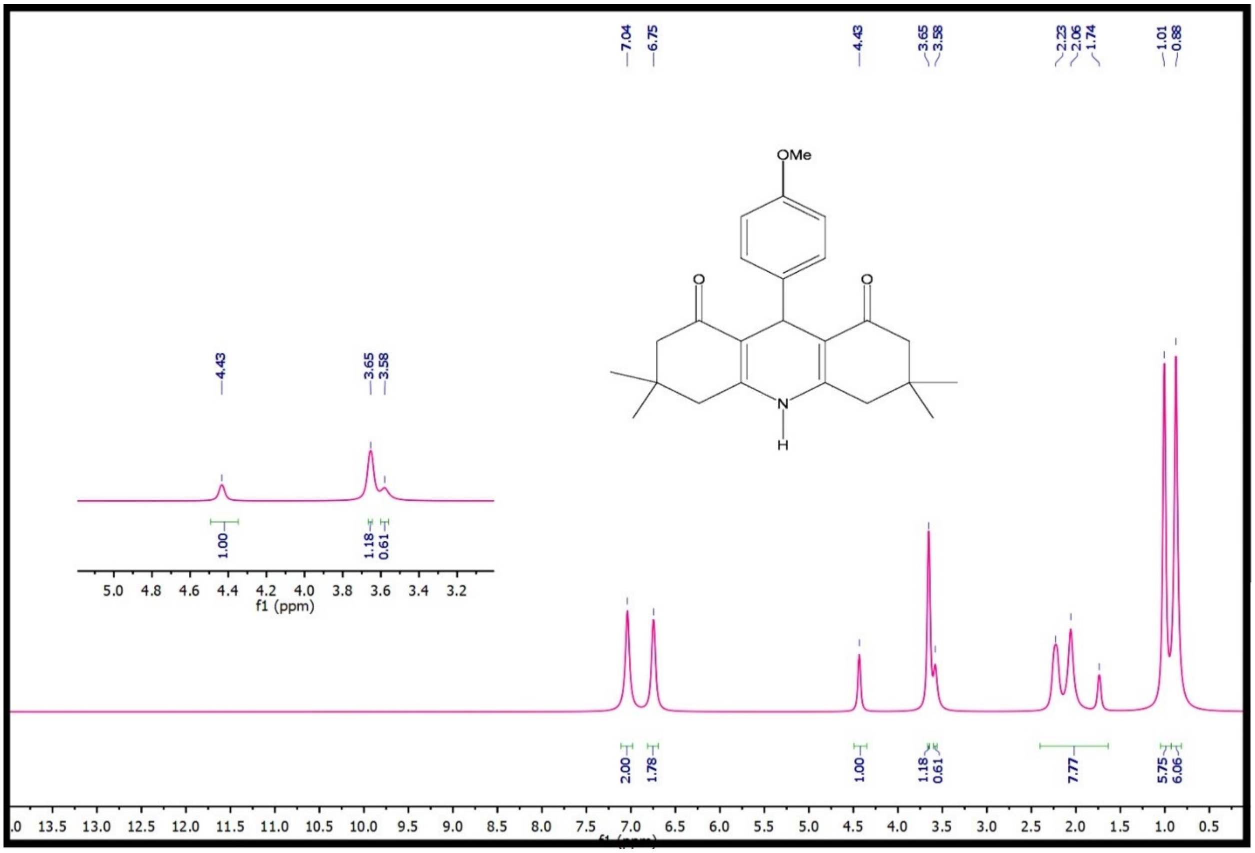

Fig.30- ^{1}HNMR products of 9-(4-methoxyphenyl)-3,3,6,6-tetramethyl-3,4,6,7,9,10-hexa-hydro-acridine -1,8-dione
